# Two Bistable Switches Govern M Phase Entry

**DOI:** 10.1016/j.cub.2016.10.022

**Published:** 2016-12-19

**Authors:** Satoru Mochida, Scott Rata, Hirotsugu Hino, Takeharu Nagai, Béla Novák

**Affiliations:** 1 Priority Organization for Innovation and Excellence, Kumamoto University, Kyoyoto-honjo 1, 2-2-1 Honjo, Chuo-ku, Kumamoto 860-0811, Japan; 2 Institute for Medical Embryology and Genetics, Kumamoto University, Kyoyoto-honjo 1, 2-2-1 Honjo, Chuo-ku, Kumamoto 860-0811, Japan; 3 International Research Center for Medical Science, Kumamoto University, Kyoyoto-honjo 1, 2-2-1 Honjo, Chuo-ku, Kumamoto 860-0811, Japan; 4 Precursory Research for Embryonic Science and Technology (PRESTO) Program, Japan Science and Technology Agency, 4-1-8 Honcho, Kawaguchi, Saitama 332-0012, Japan; 5 Department of Biochemistry, University of Oxford, South Parks Road, Oxford OX1 3QU, UK; 6 The Institute of Scientific and Industrial Research, Osaka University, Mihogaoka 8-1, Ibaraki, Osaka 567-0047, Japan

**Keywords:** mitosis, cyclin-dependent kinase, PP2A, Greatwall kinase, hysteresis, threshold, robustness

## Abstract

The abrupt and irreversible transition from interphase to M phase is essential to separate DNA replication from chromosome segregation. This transition requires the switch-like phosphorylation of hundreds of proteins by the cyclin-dependent kinase 1 (Cdk1):cyclin B (CycB) complex. Previous studies have ascribed these switch-like phosphorylations to the auto-activation of Cdk1:CycB through the removal of inhibitory phosphorylations on Cdk1-Tyr15 [1, 2]. The positive feedback in Cdk1 activation creates a bistable switch that makes mitotic commitment irreversible [2, 3, 4]. Here, we surprisingly find that Cdk1 auto-activation is dispensable for irreversible, switch-like mitotic entry due to a second mechanism, whereby Cdk1:CycB inhibits its counteracting phosphatase (PP2A:B55). We show that the PP2A:B55-inhibiting Greatwall (Gwl)-endosulfine (ENSA) pathway is both necessary and sufficient for switch-like phosphorylations of mitotic substrates. Using purified components of the Gwl-ENSA pathway in a reconstituted system, we found a sharp Cdk1 threshold for phosphorylation of a luminescent mitotic substrate. The Cdk1 threshold to induce mitotic phosphorylation is distinctly higher than the Cdk1 threshold required to maintain these phosphorylations—evidence for bistability. A combination of mathematical modeling and biochemical reconstitution show that the bistable behavior of the Gwl-ENSA pathway emerges from its mutual antagonism with PP2A:B55. Our results demonstrate that two interlinked bistable mechanisms provide a robust solution for irreversible and switch-like mitotic entry.

## Highlights

• Cdk1 auto-activation loop is dispensable for switch-like mitotic entry
• PP2A:B55 auto-regulation creates a bistable switch
• Two bistable switches provide a robust solution for mitotic entry

## Graphical Abstract

**Figure:**
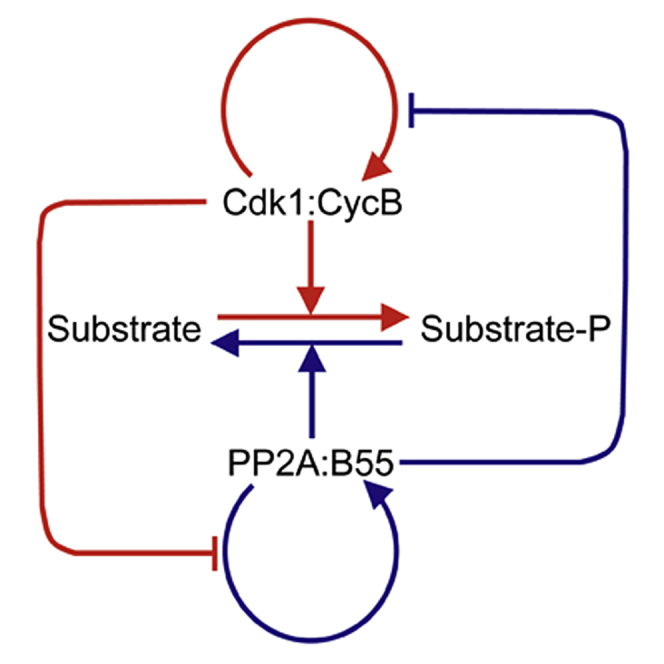

## Teaser

Mochida et al. find, using a biochemical reconstitution and mathematical simulation, that the regulation of PP2A:B55 phosphatase can create a bistable switch for mitotic phosphorylation of Cdk1 substrates. This new mechanism, together with the Cdk1 auto-activation loop, provides a robust solution for irreversible and switch-like mitotic entry.

## Results and Discussion

Cell-cycle progression requires rapid and irreversible cellular decisions at the transitions between phases. A key transition is the entry into mitosis, which requires the phosphorylation of hundreds of proteins by cyclin-dependent kinase 1 (Cdk1):cyclin B (CycB) to bring about processes such as nuclear envelope breakdown, chromosome condensation, and spindle assembly. The basic requirements of rapid and irreversible transitions are met, because the response to CycB is governed by a fast, bistable switch [2, 3, 4]. Below a CycB threshold, the cell remains in interphase, because Cdk1:CycB complexes are inactivated by phosphorylation at Cdk1-Tyr15 (Y15). Above the CycB threshold, the inhibitory Y15 phosphorylation is removed, because the Y15 kinases (Wee1/Myt1) are turned off while the Y15 phosphatase (Cdc25) is turned on [1]. Activated Cdk1 phosphorylates many mitotic substrates, including Wee1/Myt1 and Cdc25; phosphorylating these Y15-modifying enzymes makes the system auto-catalytic. However, Cdk1 also performs phosphorylations that inhibit the counteracting phosphatase PP2A:B55 that eventually removes mitotic phosphorylations at M phase exit and in interphase [5, 6, 7]. This led us to ask whether regulation of PP2A:B55 plays a role in creating the Cdk activity threshold for mitotic substrate phosphorylations.

**Figure 1:**
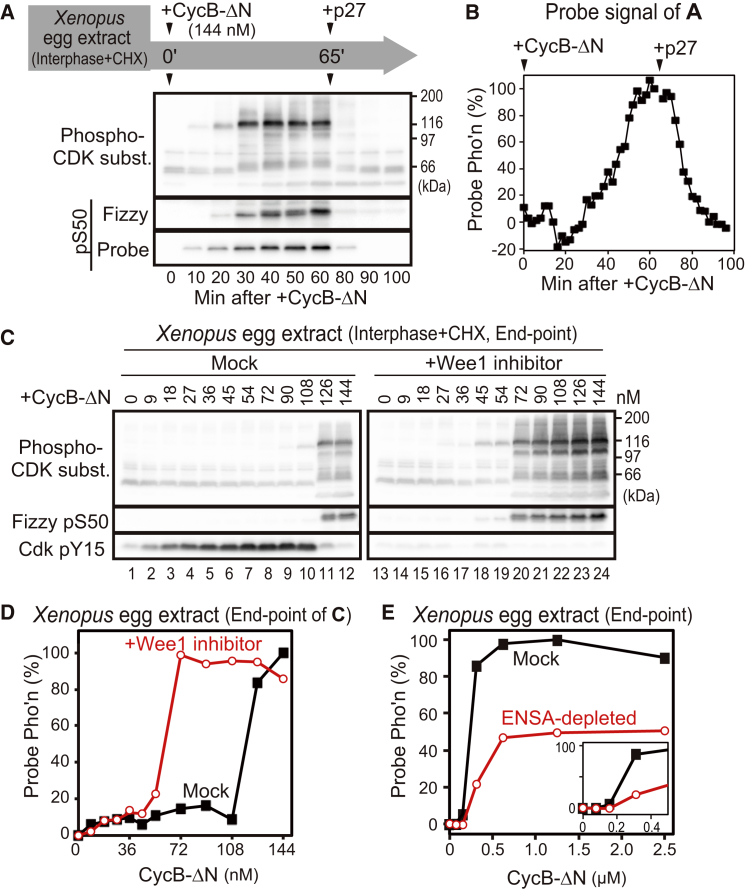
CycB Threshold of Mitotic Phosphorylation in *Xenopus* Egg Extracts Persists in the Presence of an Inhibitor of Wee1 (A) Interphase egg extract was supplemented with luminescent probe, CycB-ΔN (144 nM) and cycloheximide (CHX) at 0 min. p27^Kip1^ (450 nM) was added at 65 min for mitotic exit. Aliquots were analyzed by immunoblotting for phosphorylation (Pho’n) of CDK substrates (subst.), endogenous Fizzy-Ser50, and the probe. The probe was only weakly phosphorylated when CycB-ΔN was not added (Figure S1D). (B) Probe luminescence of (A) was plotted. (C) Interphase egg extract was supplemented with increasing amount of CycB-ΔN in the absence (left) or presence (right) of PD166285 (1 μM). After 80 min incubation, aliquots were analyzed by immunoblotting. (D) Probe luminescence of the samples shown in (C) was plotted. (E) Interphase egg extract immunodepleted of ENSA was analyzed as in (D). The inset is a magnification of the low x-axis range. Experiments were repeated three to five times, and similar results were obtained. The S50-1G4 probe was used in this figure. See also Figure S1.

We induced phosphorylation and dephosphorylation of mitotic proteins in *Xenopus* egg extracts by the addition of either non-degradable CycB (CycB-ΔN) or the Cdk inhibitor protein p27^Kip1^ (Figure 1A). To quantitatively analyze the ratio of Cdk and PP2A:B55 activities, we developed a set of luminescent probes whose light emittance reflects their phosphorylation level (Figures S1A–S1C; see also the Supplemental Experimental Procedures). The phosphorylation and light emittance of our probes correlated well with the phosphorylation/dephosphorylation cycle of mitotic proteins (Figures 1A, 1B, and S1D). We also confirmed that mitotic phosphorylations required CycB levels to be higher than a threshold (Figure 1C, Mock). At low CycB levels (at and below 108 nM), Cdk1 becomes Y15 phosphorylated (an inactive state), and both the probe and the endogenous proteins remained dephosphorylated (Figures 1C and 1D, Mock). Above a critical CycB threshold (126 nM), Cdk1 became activated by the dephosphorylation of Y15, and mitotic phosphorylations were detected (Figures 1C and 1D, Mock). We conclude that the CycB threshold of Cdk1 activation coincides with mitotic phosphorylation at the G2/M transition and that our probes can detect this threshold in *Xenopus* egg extracts.

It is well established that the CycB threshold of *Cdk1 activation* due to Y15 phosphorylation [1, 2] can be abolished by the inhibition of Wee1/Myt1 kinases [8]. Surprisingly, however, a sharp CycB threshold of *mitotic phosphorylation* persisted at a lower level of CycB (about 60 nM) when the Wee1/Myt1 inhibitor PD166285 was added to the extract, despite the lack of Y15 phosphorylation (Figures 1C and 1D). Therefore, we must distinguish two related, but not identical, thresholds: a threshold of Cdk1 activation and that of mitotic phosphorylation. Since Cdk1 activity is directly proportional to CycB levels in the absence of Cdk1-Y15 phosphorylation [8], Figures 1C and 1D indicate a Cdk1 activity threshold of mitotic phosphorylation that exists independently of the Cdk1 auto-activation mechanism.

One way to establish a Y15-independent threshold of mitotic phosphorylation would be switch-like inactivation of a Cdk1-countering phosphatase, one obvious candidate being PP2A:B55. This enzyme is inhibited by the Greatwall-kinase (Gwl)-phosphorylated form of alpha-endosulfine (ENSA) [6, 7, 9, 10, 11]. Because Gwl is activated by Cdk1 phosphorylation, Cdk1 inhibits PP2A:B55 via this Gwl-ENSA pathway, allowing the high phosphorylation level of Cdk1 substrates in mitosis [5, 12]. Therefore, we tested whether the Gwl-ENSA pathway helps establish the CycB threshold of mitotic phosphorylation in *Xenopus* egg extract. In the absence of ENSA, probe phosphorylation level showed a sigmoidal response to CycB levels with half the plateau level of the control sample, suggesting that the Cdk1 auto-activation mechanism can still operate in the absence of PP2A:B55 inhibition (Figures 1E and S1E). We conclude that the Gwl-ENSA pathway plays a major role in full, switch-like phosphorylation of mitotic substrates in response to the levels of CycB and Cdk1 activity, even when the Cdk1 auto-activation mechanism is intact.

**Figure 2:**
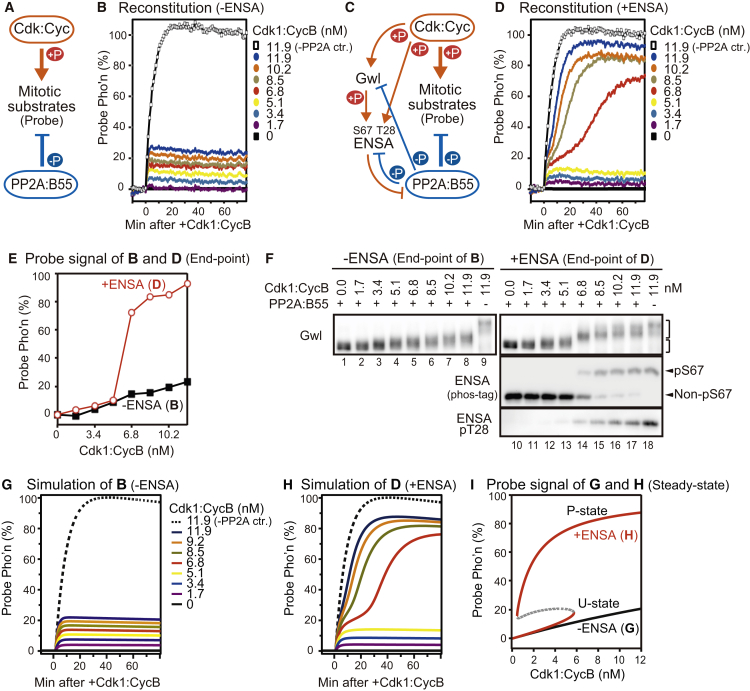
The Gwl-ENSA Pathway Creates a Cdk Activity Threshold of Mitotic Phosphorylation in a Reconstituted System (A) Simple Cdk:Cyc/PP2A:B55 antagonism. Red and blue represent mitotic and anti-mitotic effects, respectively. (B) Time-course analysis of probe phosphorylation (Pho’n) shown as in (A), with increasing Cdk1:CycB levels ([PP2A:B55] = 50 nM). A sample without PP2A:B55 was used as 100% control (ctr.; open black squares). (C) Cdk:Cyc/PP2A:B55 antagonism with the Gwl-ENSA pathway. (D) Time-course analysis of probe phosphorylation shown as in (C) ([PP2A:B55] = 50 nM, [Gwl] = 20 nM, and [ENSA] = 300 nM). (E) Endpoint analyses of (B) (black) and (D) (red). (F) Aliquots of (B) and (D) were analyzed for Gwl (top), ENSA-S67 phosphorylation (middle; upper arrowhead indicates S67-phosphorylated form of ENSA), and ENSA-T28 phosphorylation (bottom). Slower and faster migrating forms of Gwl are indicated by square brackets. Samples free from PP2A:B55 are shown in lanes 9 and 18 as fully phosphorylated controls. Representative result of five experiments is shown here. (G and H) Simulation analyses of probe phosphorylation of (B) and (D). (I) The steady states of phosphorylated probe in (G) and (H), which can be directly compared with the endpoint analysis panel in (E). The T50-NCP probe was used in this figure. See also Figures S2 and S3 and Tables S1 and S2.

To test whether the Gwl-ENSA pathway, together with Cdk1:CycB and PP2A:B55, can create a threshold by themselves in the absence of Cdk1 auto-activation, we next developed a reconstituted system of the Gwl-ENSA pathway using purified proteins (Figure S2A). As would be expected, in the absence of ENSA, the phosphorylation of both the probe and Gwl showed a graded response to Cdk1 activity with no threshold (Figures 2A, 2B, 2E, and 2F).

In the presence of the complete Gwl-ENSA pathway in the reconstitution system, Cdk1 phosphorylation of the mitotic substrates showed a switch-like response at a Cdk1:CycB concentration between 5.1 and 6.8 nM (Figures 2C–2F). The most dramatic phosphorylation shift was shown by ENSA, which was converted from the unphosphorylated to the phosphorylated form around the Cdk threshold (Figure 2F). The band shift of ENSA is caused by Gwl-catalyzed S67 phosphorylation of ENSA, which is responsible for ENSA binding to PP2A:B55 [13]. In both the presence and absence of ENSA, both the probe and Gwl are partially phosphorylated at low-Cdk activities to a similar extent, suggesting that PP2A:B55 is not inhibited below the Cdk threshold of probe phosphorylation. The sharp increase in probe phosphorylation above the Cdk threshold coincides with ENSA S67 phosphorylation, which titrates the phosphatase away from other phosphorylated substrates (e.g., both the probe and Gwl).

The existence of a Cdk threshold of mitotic probe phosphorylation in the steady-state response of the reconstituted system implies that all components of the system, including Gwl, participate in inactivating, as well as activating, reactions. If Gwl were not inactivated by PP2A:B55, there would be no Cdk threshold, since Gwl would eventually be fully activated at all Cdk activities. We therefore surmise that Gwl is a substrate of the PP2A:B55 phosphatase (Figure 2C), which keeps Gwl dephosphorylated at subthreshold Cdk activities. Furthermore, inactivation of PP2A:B55 by S67-phosphorylated ENSA (pENSA) causes a switch-like phosphorylation of Gwl above the Cdk threshold. This effect creates an antagonistic relationship between Gwl and PP2A:B55, which can produce a Cdk1 threshold for the mitotic phosphorylation [14, 15].

To better understand this antagonistic relationship, we formulated mathematical models of the Gwl-ENSA pathway (Supplemental Information). At the heart of the model is the double-negative feedback between Gwl and PP2A:B55 (Figures 2C and S2B): Gwl inhibits PP2A:B55 by activation of ENSA through S67 phosphorylation, and PP2A:B55 keeps Gwl inactive by dephosphorylating it. For completeness, we also include Gwl auto-phosphorylation, which takes place after Cdk-induced priming phosphorylation [5]. The model also contains the “unfair competition” mechanism of pENSA on the phosphatase [11]: the strong binding of pENSA to PP2A:B55 titrates the phosphatase away from all other substrates and makes itself the preferential, albeit sluggish, substrate.

The mathematical model explains the sharp transition of the Gwl-ENSA pathway from an un- or hypo-phosphorylated “U” state to a hyper-phosphorylated “P” state by a bistable switch operating at the Cdk threshold of mitotic probe phosphorylation (Figures 2G–2I and S3A–S3C). This bistable switch makes the strong and experimentally testable prediction that the system will display hysteresis. At first, the Cdk threshold for turning on the Gwl-ENSA pathway at the U→P transition (the ON threshold) must be larger than the threshold for turning it off at the P→U transition (the OFF threshold).

**Figure 3:**
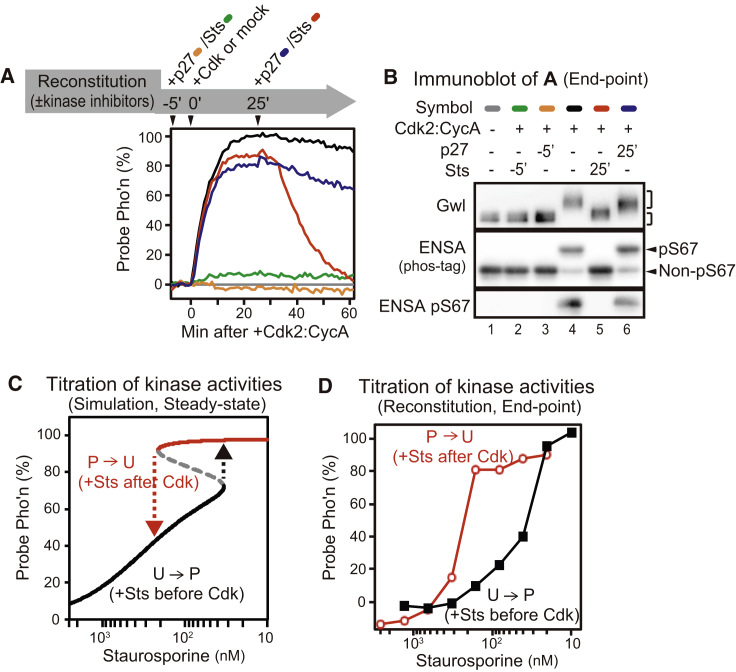
The Gwl-ENSA Pathway Defines a Bistable Switch with Two Distinct Thresholds (A) Reconstituted system was supplemented either with p27^Kip1^ (450 nM) or staurosporine (Sts) (10 μM) 5 min before or 25 min after adding a supra-threshold level of Cdk activity (20 nM of Cdk2:CycA). Pho’n, phosphorylation. (B) Endpoint analysis of the samples shown in (A) was done for Gwl and ENSA-S67 phosphorylation by immunoblotting. (C) Simulation of Gwl/PP2A:B55 antagonism with staurosporine titration suggests bistability of the reconstitution system. (D) Experimental confirmation of bistability suggested in (C). Different concentrations of staurosporine were added to the reconstituted system before (black) or after (red) the addition of a supra-threshold level of Cdk activity (20 nM of Cdk2:CycA). Data after 60 min incubation were plotted. Representative data of two experiments are shown. The S50-1G12 probe was used in this figure. See also Figures S4A–S4F and Tables S1 and S2.

We used Cdk2:CycA in the hysteresis experiments as the source of Cdk activity, because Cdk1:CycB is difficult to obtain in reasonable amounts from *Xenopus* egg extract or from insect cells. Cdk2:CycA behaves identically to Cdk1:CycB in all features in the reconstituted system, especially in producing the same Cdk threshold of mitotic probe phosphorylation (Figures S3D–S3K). p27^Kip1^ was added to the reconstituted system in the “U” and in the “P” states. Starting from the “U” state, p27^Kip1^ efficiently blocked phosphorylation of Gwl, ENSA, and the probe in the presence of a supra-threshold level (20 nM) of Cdk2:CycA (Figures 3A and 3B, orange sample). However, when p27^Kip1^ was added to the “P” state, it was insufficient to induce dephosphorylation of any of these proteins within the experimental time course (Figures 3A and 3B, blue sample). This apparent irreversibility of the Gwl-ENSA pathway in the reconstituted system is already a strong indication for a very small (close to zero) Cdk threshold, because slowing down near a threshold is a signature of bistable systems. This slow reactivation of PP2A:B55 is consistent with our model, which suggests that P→U transition requires ∼300 min after Cdk inhibition by p27^Kip1^ (Figures S4A and S4B). This long time delay of PP2A:B55 activation is caused by continued action of Gwl after Cdk inhibition by p27^Kip1^ (Figure 3B). Dephosphorylation of mitotic substrates also becomes delayed after p27^Kip1^-induced mitotic exit of *Xenopus* egg extract when the activity of PP1 is inhibited (Figure S4D), which is normally responsible for the initial inactivation of Gwl [16].

Since this increased time delay makes the estimation of the OFF Cdk threshold difficult in the reconstituted system, we decided to use a generic kinase inhibitor, staurosporine, which inhibits both Cdk and Gwl activities. 10 μM staurosporine added to the reconstituted system in the “P” state induced dephosphorylation of all proteins (Figure 3B, red sample). However, this staurosporine-induced probe dephosphorylation was also characterized by a well-defined short time delay (5 min) (Figure 3A, red line, 25–30 min). This short time delay would be the consequence of “unfair competition” between pENSA and the probe for PP2A:B55 [11, 17], which is terminated by pENSA dephosphorylation by PP2A:B55 (Figure S4C, red curve). The “unfair competition” is drastically lengthened after p27^Kip1^-induced Cdk inhibition by the mutual antagonism between Gwl and PP2A:B55 (Figures S4B and S4C, blue curves).

The mathematical model also predicts different ON and OFF thresholds when both Cdk and Gwl are inhibited by staurosporine (Figures 3C, S4E, and S4F). The hysteresis effect with staurosporine is caused by synergetic Gwl-inhibitory effects of the inhibitor and PP2A:B55 rather than different sensitivities of the two kinases to staurosporine. To test this prediction of different ON and OFF thresholds, we added exponentially increasing concentrations of staurosporine to the reconstituted system before and 25 min after the addition of 20 nM (supra-threshold) Cdk2:CycA. We then measured the steady-state level (endpoint) of probe phosphorylation (Figure 3D). 40 nM staurosporine started to suppress probe phosphorylation at the U→P transition, while 320 nM staurosporine was required to induce dephosphorylation at the P→U transition. From this result, we conclude that the reconstituted Gwl-ENSA pathway represents a bistable switch with two distinct thresholds for kinase activities.

**Figure 4:**
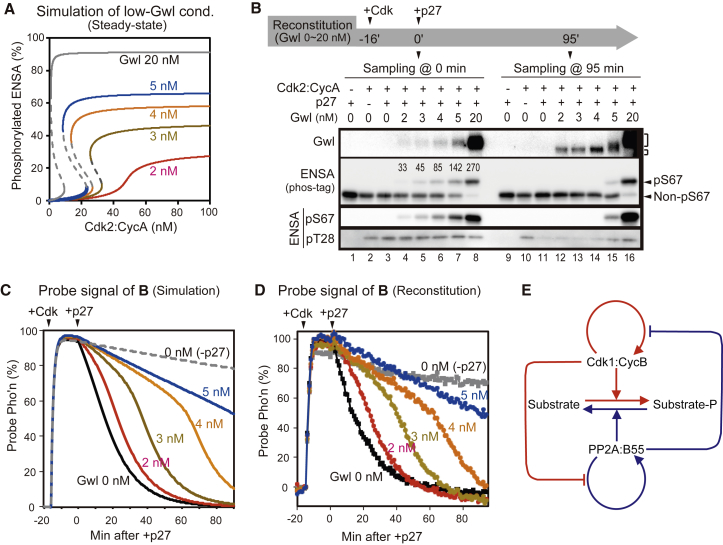
The Effect of Gwl Concentration on Cdk Thresholds and the Length of Time Delay before PP2A:B55 Reactivation (A) Steady states of the pS67 form of ENSA predicted from the model for different Gwl concentrations. cond., condition. (B) Model in (A) was experimentally tested in the reconstituted system. Samples of lower Gwl (0–20 nM) and higher Cdk (100 nM of Cdk2:CycA) concentrations were incubated for 16 min. Aliquots of samples were taken before (0 min, left) and 95 min after (right) the addition of p27^Kip1^. Numbers in the ENSA blot (phos-tag, second from top) indicate concentrations of S67-phosphorylated ENSA in nanomolar (nM). PP2A:B55 is 50 nM. The effect of ENSA T28 phosphorylation is described in Figure S4 and the Supplemental Information. (C and D) Simulation (C) and experimental (D) analyses of the time course of probe phosphorylation (Pho’n) shown in (B) were done. Representative data of four experiments are shown. (E) Schematic diagram of two interlinked bistable switches. The S50-1G12 probe was used in this figure, including the model. See also Figures S4G and S4H and Tables S1 and S2.

We next asked whether we could obtain further evidence for the hysteresis of the reconstituted Gwl-ENSA pathway by manipulating the concentrations of Gwl and Cdk to decrease the time delay after the addition of p27^Kip1^. Our model predicts that both the ON and OFF Cdk thresholds of mitotic phosphorylation are increased with the lowering of Gwl concentration (Figure 4A), and an increase of the OFF threshold provides an opportunity to decrease the time delay after Cdk inhibition by p27^Kip1^. Intuitively, at low-Gwl concentration, more Cdk is required to turn on the system (U→P), but it switches off faster after Cdk inhibition (P→U).

To test this prediction, we induced the “P state” at low Gwl levels using a supra-threshold concentration (100 nM) of Cdk2:CycA in the reconstitution system for 16 min before adding p27^Kip1^ (time = 0 in Figure 4B). S67 phosphorylation of ENSA just before p27^Kip1^ addition was increased with increasing Gwl levels, reaching stoichiometric balance with PP2A:B55 around 3 nM Gwl concentrations (Figure 4B, lanes 4–8). As we expected, phosphorylated ENSA and phosphorylated Gwl disappeared 95 min after the addition of p27^Kip1^ in the samples containing Gwl at 2–4 nM, an indication of P→U transition (Figure 4B, lanes 12–14). To get more insights about this experiment, we analyzed probe dephosphorylation of the same set of samples. As expected from the model, increasing Gwl concentrations delayed probe dephosphorylation in the reconstituted system, with a strikingly biphasic response at higher Gwl concentrations (Figures 4C and 4D). Assuming that Cdk inhibition is complete after p27^Kip1^ addition, the curves of Figure 4D allowed us to calculate the time course of release of PP2A:B55 from ENSA (Figure S4G; see the Supplemental Information). These calculations show a relatively abrupt activation of PP2A:B55 from pENSA inhibition, which makes the dephosphorylation of the probe biphasic. Since the model prediction matched well with the experiment (Figure S4H), this result further supports the idea that the reconstituted pathway has hysteresis.

Our work identifies the Gwl-ENSA-PP2A:B55 pathway as a bistable switch in the reconstituted system and also as a source of the Cdk1 threshold of mitotic phosphorylation in *Xenopus* egg extracts. The present work, together with the existence of the Cdk1 auto-activation loop through Y15 phosphorylation, led us to conclude that the eukaryotic mitotic control system is equipped with two bistable switches (Figure 4E). These two systems mutually inhibit each other to ensure that the two switches occupy opposite states (Cdk1 ON, PP2A:B55 OFF and vice versa). This mutual regulation is dictated by PP2A:B55 dephosphorylation of Cdk1-Y15-modifying enzymes (Wee1 and Cdc25 [18]) and by Cdk1 phosphorylation of Gwl [5, 19]. The two interlinked switches create a robust solution for switch-like mitotic substrate phosphorylation. This robustness is underlined by our results showing that the Gwl-ENSA-PP2A:B55 pathway can by itself maintain switch-like mitotic entry, even when Cdk1-Y15 phosphorylation is compromised; a similar situation can also be observed in proliferating fission yeast [20].

Having two interlinked bistable switches regulating the transitions between interphase and mitosis enhances the difference between the ON and OFF Cdk1 thresholds, which makes state transitions more challenging. Therefore, additional components (“triggers”) might facilitate the transitions of the switch system from one stable state to the other. CycA-dependent kinases have been suggested as triggers for mitotic entry, as they have considerable kinase activity in interphase [21, 22], consistent with our observation that Cdk2:CycA can activate the Gwl-ENSA pathway. On mitotic exit, in contrast, dephosphorylation of Gwl by PP1 [16, 23, 24] could initiate the reverse transition by supporting the PP2A:B55 auto-activation. Whether the design principle of interlinked toggle switches with a pair of triggers is a generic feature of other decision-making pathways in living cells is a question for future experimental and modeling studies.

## Author Contributions

S.M. and B.N. designed the experiments, which S.M. performed. B.N. and S.R. performed mathematical analyses. S.M., H.H., and T.N. developed luminescent probes. B.N., S.M., and S.R. wrote the manuscript.

## Supplemental Information

Document S1. Supplemental Experimental Procedures, Figures S1–S4, and Tables S1 and S2

Document S2. Article plus Supplemental Information
