# Supplementary material for: Two Bistable Switches Govern M Phase Entry
Source: Curr Biol. 2016 Dec 19;26(24):3361–7. doi: 10.1016/j.cub.2016.10.022 (PMC5196020; doi:10.1016/j.cub.2016.10.022)
Supplement: Document S1. Supplemental Experimental Procedures, Figures S1–S4, and Tables S1 and S2 [file mmc1.pdf]

Current Biology, Volume *26*

Supplemental Information

Two Bistable Switches Govern M Phase Entry

Satoru Mochida, Scott Rata, Hirotsugu Hino, Takeharu Nagai, and Béla Novák

Supplemental Figures

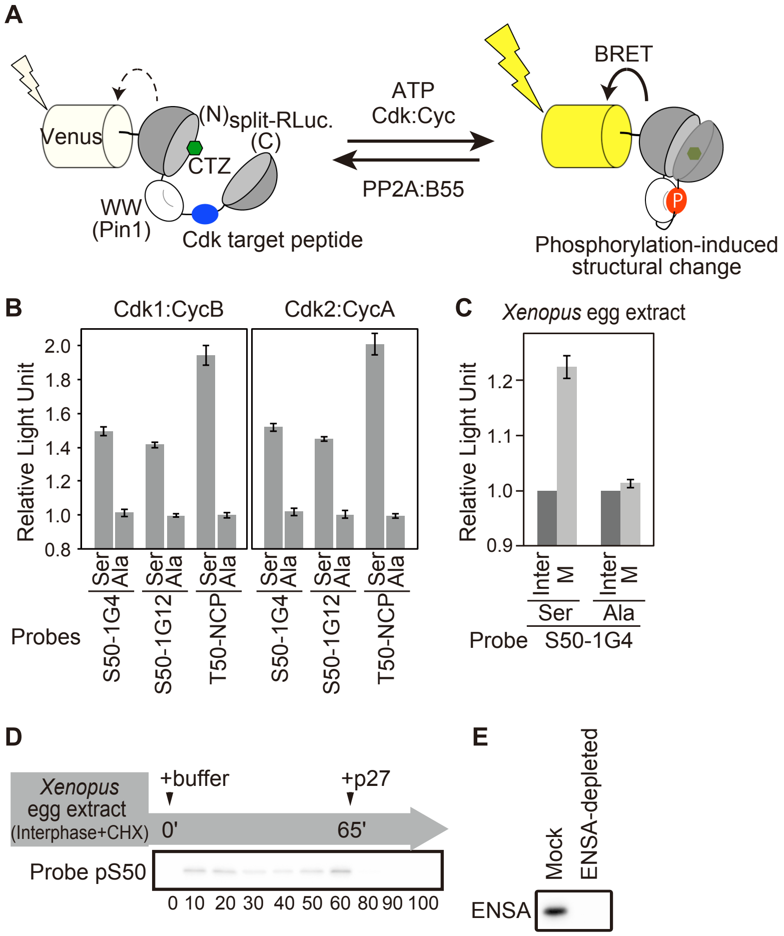

Figure S1 (Related to Figure 1): Development and analyses of the luminescent probes in purified and *Xenopus* egg extract systems. (**A**) Schematic representation of the probes [S1]. The WW domain of Pin1 and CDK-target peptide sequence were inserted into *Renilla reniformis* luciferase (RLuc). Physical interaction of WW domain with the phosphorylated peptide causes a reconstitution of the split RLuc, resulting in higher light emittance. Luciferase substrate coelenterazine-h (CTZ) is shown in green. See Methods for more details. (**B**) Probes used in this study were *in-vitro* phosphorylated by Cdk1:CycB (left) or by Cdk2:CycA (right) complexes. Luminescence 40 minutes after incubation at 30°C were plotted in relative light unit with standard deviation of three separate experiments. Probes with alanine mutation (Ala) at the phosphorylation site did not show any significant change of luminescence. (**C**) Interphase *Xenopus* egg extracts were supplemented either with S50-1G4 (Ser) or S50A-1G4 (Ala) probes. CycB-ΔN (120 nM) was added to induce mitotic entry at 23°C and luminescence after 80-minutes incubation were plotted with standard deviations of three separate experiments. (**D**) Sample without CycB-ΔN addition in Fig. 1A was analysed by immunoblotting for probe phosphorylation. (**E**) Efficiency of ENSA immunodepletion of the samples shown in Fig. 1E.

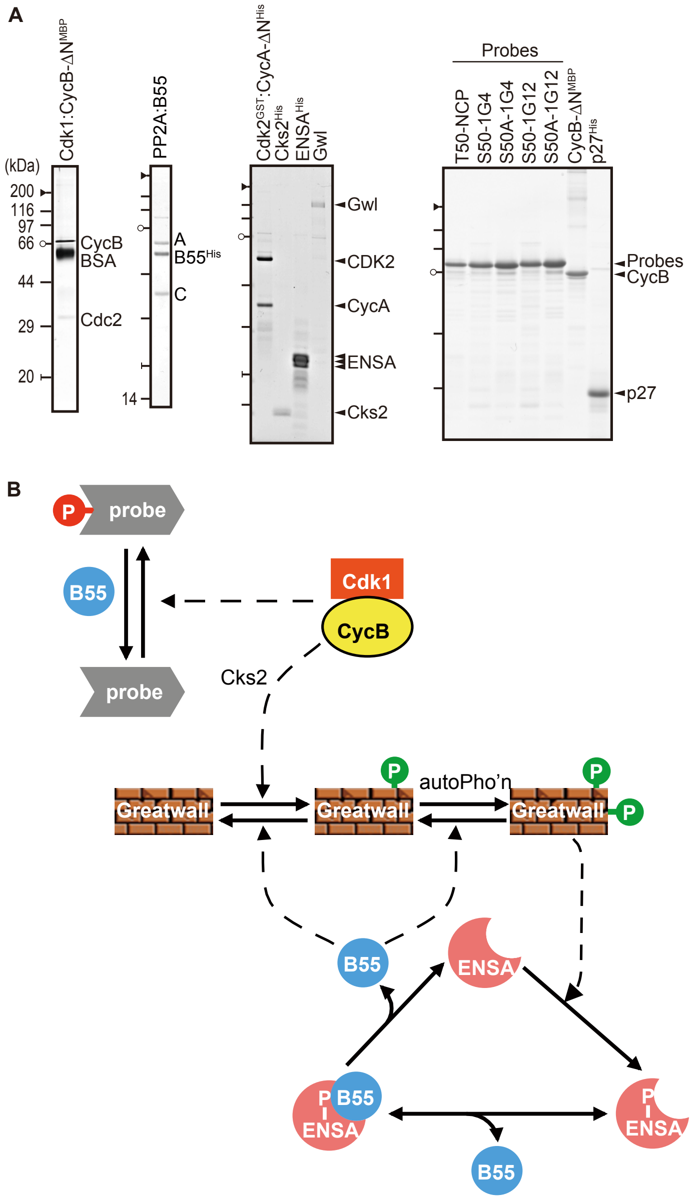

Figure S2 (Related to Figure 2): Biochemical reaction mechanism of the system reconstituted using purified proteins. (**A**) Purified protein samples separated on SDS-PAGE were either silver-stained (Cdk1:CycB) or stained with Coomassie brilliant blue. Positions of molecular weight markers are shown on the left. Arrowheads indicate positions of proteins of interest. BSA: bovine serum albumin. (**B**) This wiring diagram has been converted into differential equations of the model. Solid and dashed arrows represent chemical reactions and catalytic activities, respectively. The priming-phosphorylation of Gwl is catalysed by Cdk:cyclin complex. Cdk-activated Gwl undergoes auto-phosphorylation [S2] and the double-phosphorylated form of Gwl activates ENSA. Gwl-phosphorylated ENSA binds to PP2A:B55 and undergoes enzymatic dephosphorylation by the phosphatase. Both phosphorylations of Gwl are counter-acted by PP2A:B55 not bound to pENSA, which is the only phosphatase in our reconstituted system. Phosphorylation/dephosphorylation of the probe by Cdk:cyclin and PP2A:B55 happens downstream of the double-negative feedback between Gwl and PP2A:B55. For simplicity, T28-phosphorylation/dephosphorylation of ENSA by Cdk and PP2A:B55 are not indicated.

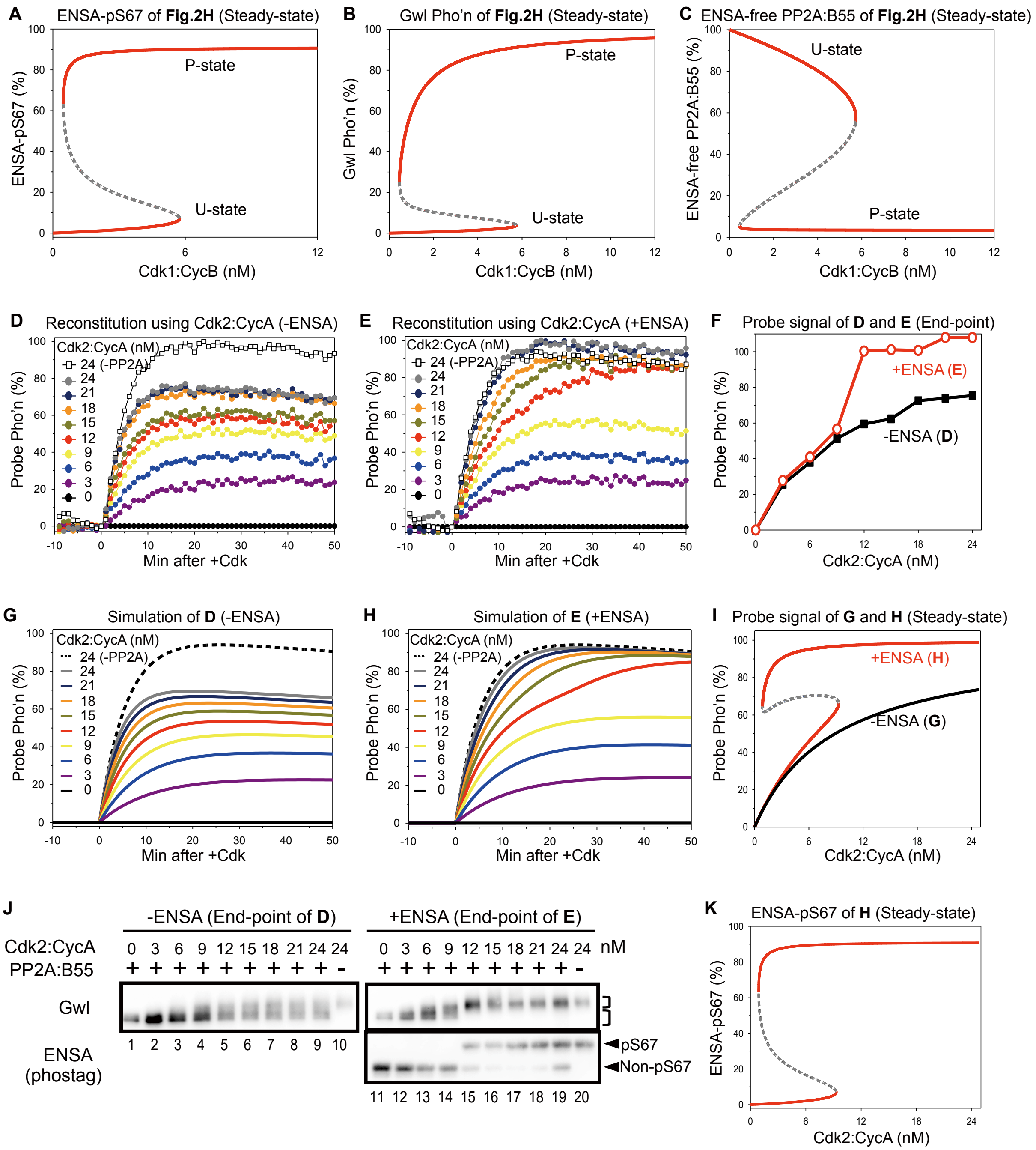

Figure S3 (Related to Figure 2, Tables S1 and S2): Kinetic modeling of the experiment in Figure 2 with Cdk1:CycB (A-C) and reproduction of Figure 2 using Cdk2:CycA complex (D-K). Model-calculated steady states of ENSA-pS67 (**A**), pGwl (**B**) and ENSA-free PP2A:B55 (**C**) are plotted for the experiment on Fig. 2. The edges of the upper and the lower steady states of the one-parameter (Cdk1:CycB) bifurcation diagrams represent Cdk1 activity thresholds where the system undergoes an abrupt state transition between unphosphorylated (U) and phosphorylated (P) states. **(D-K)** Phosphorylation of S50-1G12 probe was induced with indicated concentrations of Cdk2:CycA in the absence (**D** and **G**) and in the presence (**E** and **H**) of ENSA. Note that this probe is 7-times less sensitive to PP2A:B55 than T50-NCP probe as shown in Table S2, resulting in relatively higher phosphorylation level in all samples than those in Fig. 2. Experimental data (**D**, **E**, **F**) and calculations with the model (**G**, **H**, **I**) are shown. Probe phosphorylation is expressed relative to the maximal phosphorylation achieved by omitting PP2A:B55

(lanes 10 and 20 in **J** of this figure, otherwise 50 nM) from the reconstitution system. The end-points of probe phosphorylation (**F**) in the experiments are matched by the stable steady states of the model (**I**). (**J**) Western-blot analysis of Gwl and ENSA phosphorylations at the end-point of panels **D** and **E**. The slower-migrating form of ENSA is Ser67-phosphorylated. (**K**) One-parameter bifurcation diagram for ENSA-pS67. Representative result of 6 experiments is shown here.

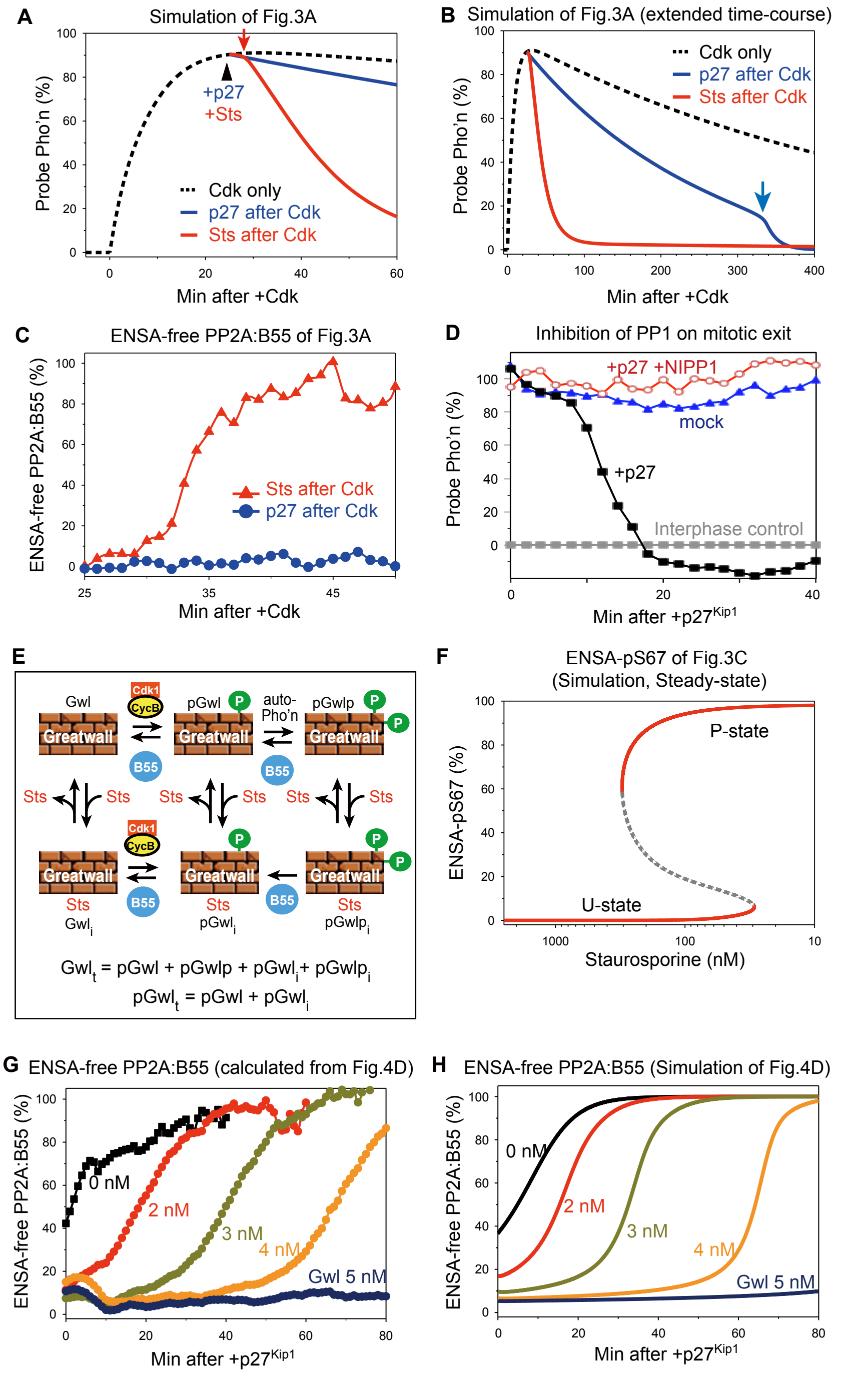

Figure S4 (Panels A-F and G-H are related to Figure 3 and 4, respectively. Also related to Tables S1 and S2): The effect of protein-kinase inhibitors. (**A**) Numerical simulation of protein-kinase inhibition in the P-state (corresponds to Fig. 3A) by p27 (blue curve) and Staurosporine (red curve). Kinase inhibition was implemented at 25 mins (blue and red curves) and Staurosporine concentration was set to 10 μM. (**B**) Extended time-course simulation of **A**. Timings of PP2A:B55 reactivation are indicated by colored arrows in **A** and **B**. (**C**) The release of PP2A:B55 from ENSA inhibition after protein-kinase inhibition by p27 and Staurosporine at 25 mins in the experiment of Fig. 3A. Observe the time-delay in PP2A:B55 release after Staurosporine addition caused by the 'unfair competition' mechanism. (**D**) PP1 activity is required for dephosphorylation of Cdk1 substrate on mitotic exit in *Xenopus* egg extract. (**E**) Wiring diagram to explain the extension of the model to the effect of Staurosporine (Sts) on Gwl. Staurosporine reversibly binds to all forms of Greatwall and thereby inhibits both auto-

phosphorylation and phosphorylation of ENSA. (**F**) Steady state dependence of ENSA S67-phosphorylation on Staurosporine concentration (increasing from the right to left). About ten-times more concentration of Staurosporine is required to induce the P→U transition than to block the U→P transition. (**G, H**) Release of PP2A:B55 from the pENSA:PP2A:B55 complex after Cdk inhibition with p27. (**G**) Calculation of ENSA-free PP2A:B55 from experimental data of probe dephosphorylation in Fig. 4D. The high concentration of Cdk2:CycA complex (100 nM) in this experiment causes significant T28-phosphorylation of ENSA, which reduces the level of ENSA-free PP2A:B55 at 0 min even in the absence of Gwl (black). The time-delay of PP2A:B55 release from ENSA inhibition is longer at higher Gwl concentrations. (H) Model simulations of **G**.

Table S1: Kinetic parameters used in the model. Related to Figures 2, 3, 4, S3, S4 and Table S2.

Parameter	Description	Value
Cdk	Cdk1:CycB or Cdk2:CycA complex	0 – 100 nM
Sub_T	Total concentration of probe	50 nM
$k_{Cdk,Sub}$	Probe phosphorylation by Cdk:Cyc complexes	see Table S2
$k_{B55,Sub}$	Probe dephosphorylation by PP2A:B55	see Table S2
$k_{d,Sub}$	Probe decay for T50-NCP and S50-1G12	0.001 min^{-1} and 0.002 min^{-1}
$ENSA_T$	Total concentration of ENSA	300 nM
$B55_T$	Total concentration of PP2A:B55	50 nM
$k_{Gw,ENSA}$	ENSA phosphorylation by active Greatwall	0.2358 $nM^{-1}min^{-1}$
k_{ass}	Association of pS67-ENSA and PP2A:B55	0.3350 $nM^{-1}min^{-1}$
k_{diss}	Dissociation of the pS67-ENSA:PP2A:B55 complex	0.0267 min^{-1}
k_{cat}	Dephosphorylation of pS67-ENSA by PP2A:B55	2.7504 min^{-1}
Gwl_T	Total concentration of Greatwall	20 nM
$k_{Cdk,Gwl}$	Greatwall phosphorylation by Cdk1:CycB or Cdk2:CycA	0.0023 or 0.0012 $nM^{-1}min^{-1}$
$k_{B55,Gwl}$	Greatwall dephosphorylation by PP2A:B55	0.1447 $nM^{-1}min^{-1}$
α	Equilibrium of Gwl de- and auto-phosphorylation	0.003 nM^{-1}
$k_{Cdk,ENSA}$	ENSA phosphorylation by active Cdk:Cyc complex	0.0016 $nM^{-1}min^{-1}$
$k_{ass,T}$	Association of pT28-ENSA and PP2A:B55	1.6068 $nM^{-1}min^{-1}$
$k_{diss,T}$	Dissociation of pT28-ENSA:PP2A:B55 complex	145.256 min^{-1}
$k_{cat,B55T}$	Dephosphorylation of pT28-ENSA by PP2A:B55	0.5211 min^{-1}

Table S2: Phosphorylation and dephosphorylation rate constants for luminescent probes. Related to Figures 2, 3, 4, S3, S4 and Table S1.

Name of probe	$k_{Cdk,Sub}$ (nM^{-1}min^{-1})	$k_{B55,Sub}$ (nM^{-1}min^{-1})
T50-NCP	0.010	0.0090
S50-1G12	0.0073	0.0013
S50-1G4	0.0235	0.017

Supplemental Experimental Procedures

Interphase *Xenopus* cell-free egg extracts

Cytostatic factor-arrested extract prepared from unfertilized *Xenopus* eggs [S3] was released into interphase for 40 mins at 23°C by addition of $CaCl_2$ (0.4 mM) in the presence of cycloheximide (0.1 mg/mL). Extract was stored in liquid nitrogen.

Immunodepletion

Immunodepletion of ENSA from egg extracts was done as described before, except use of protein-A Sepharose beads [S4].

Antibodies and chemicals

Anti-phosphoSer Cdk targets and -GFP antibodies were purchased from Cell Signaling Technology (#2324) and Medical & Biological Laboratories (clone 1E4), respectively. Anti-phosphoSer50 Fizzy and -phosphoTyr15 Cdc2 antibodies were generous gifts from Drs. Tim Hunt and Julian Gannon (Crick Institute, UK). Anti-Gwl, -ENSA, -phosphoS67-ENSA, and -phosphoT28-ENSA antibodies were previously described [S5, 6]. Coelenterazine-h, PD166285 dihydrochloride, Staurosporine and phos-tag were purchased from Wako (#035-22991), R&D SYSTEMS (#3785/1), LC Laboratories (#S-9300) and NARD (AAL-107), respectively.

Recombinant protein

Polyhistidine-tagged *Xenopus* ENSA, human Cks2, $p27^{Kip1}$, and luminescent probes were expressed and purified from bacteria strain BL21(DE3) codon+(PR) using HisPur Ni-NTA Resin (Thermo Scientific) according to the manufacturer's protocol. GST-tagged *Xenopus* Greatwall was expressed in and purified from Sf9 insect cells as described previously [S7] and GST portion was removed by using HRV-3C protease. The Aα, B55δ, and Cα subunits of PP2A complex were simultaneously expressed in HighFive insect cells and purified as described [S4]. Cdk2:CycA complex was prepared as described [S8]. Cdk1:CycB complex was purified as described [S9] with some modifications as follows. Maltose-binding protein-tagged human CycB protein lacking 172 amino acids of its amino terminus (CycB-ΔN) was expressed and purified from bacteria by using amylose resin (New England Biolab). CycB-ΔN protein was incubated with interphase egg extract in the presence of PD166285 (1 μM) for 30 mins at 23°C. Cdk:Cyc complexes were purified by using Suc1 beads, and eluate from the beads was further purified using amylose resin. All proteins were dialyzed against a storage buffer (20 mM Tris-HCl, 150 mM NaCl, 0.01% Tween-20, 0.1 mM dithiothreitol (DTT), pH7.5) and stocked in small aliquots at -80°C. Unless otherwise mentioned, each protein concentration in the reconstitution system was as follows: Cks2=200 nM, Gwl=20 nM, ENSA=300 nM, PP2A:B55=50 nM. Our rough estimates of endogenous concentrations of these proteins are as follows: Gwl=17~21 nM, ENSA=200~300 nM, PP2A:B55=50~70 nM.

Luminescent probe assay

Luminescent phosphorylation probes, consisting of engineered luminescent protein called "NanoLantern" inserted just after its Gly228 residue with the phosphopeptide-binding WW domain of Pin1 and a modified ~30-amino-acid-long peptide derived from *Xenopus* Fizzy containing its Ser50 residue (Fig. S1A), were developed to detect the ratio of Cdk and PP2A:B55 activities [S1]. We used 3 probes with different phosphorylation sequences as follows: Probe S50-1G4: (N')-RSAYMMGGRRVSANTSTL*SPMKASNRSHSSSGGLE-(C'), S50-1G12: (N')-RSGGGCSSLNTSANTSTL*SPMKASNYSHRNAYELE-(C'), T50-NCP: (N')-RSGGGRAEKKKPANTSTL*TPMKASNTKQAKKGGVE (C'). Asterisk denotes each phosphorylation site. Serine/Threonine-to-Alanine mutants of the probes were also made (Figs S1B, C). Purified probe (50 nM) was mixed with an appropriate combination of other proteins in a reconstitution buffer (20 mM Tris-HCl, 50 mM NaCl, 5 mM $MgCl_2$, 7.5 mM KCl, 10 μM $MnCl_2$, 0.01% Tween-20, 1 mM DTT, 20 mM ascorbic acid, 0.5 mM adenosine triphosphate, 50 μg/mL bovine serum albumin, 10 μM Coelenterazine-h (CTZ), pH7.5). Assays were started in white-wall 96-well microplate (#4ti-0961, 4titude) at 30°C, and luminescence was measured using Infinite F200 Pro microplate reader (TECAN). Assays in egg extracts contained a probe at 200 nM and Coelenterazine-h at 10 μM. All data were standardized by using samples without Cdk in the reconstitution system or without addition of CycB in egg extracts as baseline controls.

SDS-PAGE analyses with phos-tag reagent and quantification of immunoblot data

To quantitate S67-phosphorylated form of ENSA, standard SDS polyacrylamide gel was supplemented with 15 µM of phos-tag reagent, which causes slower migration of phosphorylated proteins on electrophoresis. Under this condition, ENSA phosphorylation on its S67, but not on T28, significantly decreases its mobility and resulted in an up-shifted band [S6]. All immunoblot signals were acquired using a FUSION SOLO S system (VILBER LOURMAT). In Fig.4B, signal intensities of the up-shifted bands were quantified using the Fusion Capt Advance software. For better quantification, a tendency of CDK-phosphorylated ENSA to react less with our anti-ENSA antibody was also considered (Compare the lanes 1 and 2 of Fig.4B).

Mathematical modeling of the Greatwall-ENSA pathway

We used nonlinear ordinary differential equations (ODEs) to describe the dynamics of the Gwl-ENSA pathway. The ODEs describe the time-rate of change of the components in the pathway using the law of mass action for each elementary reaction. Each enzymatic reaction (except pENSA dephosphorylation by PP2A:B55) is approximated as a bimolecular, second-order reaction, thereby ignoring the enzyme-substrate complexes. This approximation is reasonable if the Michaelis-constants (K_M) of the enzymes are larger than the substrate concentrations, in which case the reaction is always first-order for the substrate. This assumption is experimentally validated for phosphorylation and dephosphorylation of the probes with purified Cdk:cyclin and PP2A:B55 complexes, respectively. This simplifying assumption had to be waived in the case of pENSA dephosphorylation by PP2A:B55 because of their tight binding (small K_M value).

Cdk:cyclin complex phosphorylates Gwl at site T193 (pGwl), which activates the intramolecular autophosphorylation of Gwl into its double-phosphorylated form:

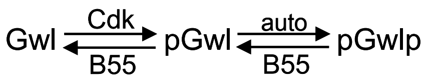

Both phosphorylation reactions are counter-acted by PP2A:B55 (B55)-catalysed dephosphorylations, because PP2A:B55 is the only phosphatase in the system. Since the total Gwl concentration (Gwl_T) is constant (conservation condition), Gwl dynamics can be described by two ODEs:

$$\frac{d[pGwl]_t}{dt} = k_{Cdk,Gw} \cdot [Cdk] \cdot ([Gwl]_T - [pGwl]_t) - k_{B55,Gw} \cdot [B55] \cdot ([pGwl]_t - [pGwlp]) \qquad (1')$$

$$\frac{d[pGwlp]}{dt} = k_{auto} \cdot ([pGwl]_t - [pGwlp]) - k'_{B55,Gw} \cdot [B55] \cdot [pGwlp] \qquad (2)$$

where pGwlt is the sum of the two phosphorylated forms (pGwlt = pGwl + pGwlp). Our parameter estimation suggests that the second ODE is very fast, therefore we assume that pGwl and pGwlp are in pseudo-steady states:

$$[pGwlp] = \frac{[pGwl]_t}{1 + \alpha \cdot [B55]} \qquad [pGwl] = = \frac{\alpha \cdot [B55] \cdot [pGwl]_t}{1 + \alpha \cdot [B55]} \qquad (3)$$

where α indicates the ratio of $k'_{B55,Gw}$ and k_{auto} rate constants of the second step. With this simplification, Eq.(1') takes the following form:

$$\frac{d[pGwl]_t}{dt} = k_{Cdk,Gw} \cdot [Cdk] \cdot ([Gwl]_T - [pGwl]_t) - k_{B55,Gw} \cdot \alpha \cdot [B55]^2 \cdot \frac{[pGwl]_t}{1 + \alpha \cdot [B55]} \qquad (1)$$

In the model, ENSA is phosphorylated at the S67 site (pENSA) by auto-phosphorylated Greatwall-kinase (pGwlp), which is the only form that catalyses ENSA phosphorylation. pENSA binds rapidly and tightly to free PP2A:B55, which dephosphorylates it:

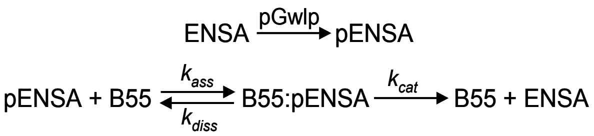

Considering that total ENSA concentration ($[ENSA]_T$) in our reconstituted system is constant, we derive an ODE for the sum of the two phosphorylated forms ($[pENSA]_t$ = [pENSA] + [B55:pENSA]):

$$\frac{d[pENSA]_t}{dt} = k_{Gw,ENSA} \cdot [pGwlp] \cdot ([ENSA]_T - [pENSA]_t) - k_{cat,B55} \cdot [B55{:}pENSA] \qquad (4)$$

and a second ODE for PP2A:B55-pENSA complex:

$$\frac{d[B55:pENSA]}{dt} = k_{ass} \cdot [B55] \cdot ([pENSA]_t - [B55:pENSA]) - (k_{cat,B55} + k_{diss}) \cdot [B55:pENSA] \quad (5)$$

The free PP2A:B55 (B55) concentration is calculated from the conservation condition that the total concentration ($B55_T$) is constant:

$$[B55] = [B55]_T - [B55:pENSA] \quad (6)$$

Finally, the probe (Sub) carrying a Fizzy phosphorylation site is interconverted by Cdk:cyclin and PP2A:B55 complexes between phosphorylated (pSub) and unphosphorylated forms (Sub_T – pSub):

$$\frac{d[pSub]}{dt} = k_{Cdk,Sub} \cdot [Cdk] \cdot ([Sub]_T - [pSub]) - \left(k_{B55,Sub} \cdot [B55] + k_{d,Sub}\right) \cdot [pSub] \quad (7)$$

Although the total concentration of the probe is constant, it slowly loses its light emittance, which we approximate with a slow exponential decay ($k_{d,Sub}$):

$$\frac{d[Sub]_T}{dt} = -k_{d,Sub} \cdot [Sub]_T \quad (8)$$

Cdk2 inhibition by p27 is simulated by setting the Cdk parameter to zero, because the inhibitor is used in large excess (> 450 nM) over the kinase.

This system of differential- and algebraic-equations which was solved by numerical integration, provides a comprehensive picture of temporal changes in our reconstitution experiments. The temporal evolution of the biochemical system leads to a steady state which corresponds to the end-point of the experiments. The steady state solutions of the system are conveniently characterized by bifurcation diagrams, where one of the dynamical variables is plotted on the y-axis as a function of Cdk:cyclin complex, which is our experimentally controlled parameter.

The following code can be used with freely available XPPAut software (http://www.math.pitt.edu/~bard/xpp/xpp.html) to reproduce our figures of temporal simulations (Fig. 2G,H, 4C, S3G,H, and S4H). Since ENSA becomes phosphorylated directly by Cdk:cyclin complexes at the T28 site, our code also includes this reaction, which becomes significant when the ratio of Cdk:cyclin to Gwl is very large. The two independent phosphorylations result in three different phosphorylated forms of ENSA (pENSA, ENSAp and pENSAp) indicated on the left (S67) and on the right (T28), respectively. T28-ENSA phosphorylation is also removed by PP2A:B55, at least in our system.

```
# .ode file for time-course simulation of the reconstituted system
dSubT/dt = - kdSub*SubT
dpSub/dt =  kCdkSub*Cdk*(SubT-pSub) - (kB55Sub*B55 +  kdSub)*pSub
dpENSA/dt = kGwENSA*pGwlp*ENSA + kdiss*B55pENSA - kass*pENSA*B55 - kCdkENSA*Cdk*pENSA
+ kcatB55T*pENSApB55
dB55pENSA/dt = kass*pENSA*B55 - kdiss*B55pENSA - kcatB55*B55pENSA
dpENSAp/dt = kCdkENSA*Cdk*pENSA + kdissT*pENSApB55 - kassT*pENSAp*B55 +
kGwENSA*pGwlp*ENSAp + kdiss*B55pENSAp - kass*B55*pENSAp
dpENSApB55/dt = kassT*pENSAp*B55 - kdissT*pENSApB55 - kcatB55T*pENSApB55
dB55pENSAp/dt = kass*pENSAp*B55 - kdiss*B55pENSAp - kcatB55*B55pENSAp
dENSAp/dt = kcatB55*B55pENSAp - kassT*ENSAp*B55 + kdissT*ENSApB55 - kGwENSA*pGwlp*ENSAp
+ kCdkENSA*Cdk*ENSA
dENSApB55/dt = kassT*B55*ENSAp - kdissT*ENSApB55 - kcatB55T*ENSApB55
dpGwlt/dt = kCdkGw*Cdk*(GwlT - pGwlt) - kB55Gw*alfa*B55^2*pGwlt/(1 + alfa*B55)
pGwlp = pGwlt/(1+alfa*B55)
B55 = B55T - B55pENSA - pENSApB55 - B55pENSAp - ENSApB55
ENSA = ENSAtot - pENSA - B55pENSA - pENSAp - pENSApB55 - B55pENSAp - ENSAp - ENSApB55
aux S67pENSA = pENSA + B55pENSA + pENSAp + B55pENSAp + pENSApB55
aux pGwlp = pGwlt/(1 + alfa*B55)
aux B55free = B55T - B55pENSA - pENSApB55 - B55pENSAp - ENSApB55
init SubT=50, pSub=0, pGwlt=0

par Cdk=0
# kinetic parameters for Cdk1:CycB and T50-NCP probe:
par kCdkSub=0.01, kB55Sub=0.009, kdSub=0.001
par ENSAtot=300, B55T=50, kGwENSA=0.2358
par kass=0.3350, kdiss=0.0267, kcatB55=2.7504
par GwlT=20, kCdkGw=0.0023, kB55Gw=0.1447, alfa=0.003
par kCdkENSA=0.0016, kassT=1.6068, kdissT=145.256, kcatB55T=0.5211
# for Cdk2:CycA and S50-1G12 probe set:
# kCdkSub=0.0073, kB55Sub=0.0013, kdSub=0.002, kCdkGw=0.0012
@ total=50,dt=0.5,meth=STIFF,xp=time,yp=pSub,xlo=0,xhi=50,ylo=0,yhi=50
@ NTST=15,NMAX=1000000,NPR=10000,DS=0.01,BOUNDS=2000
@ DSMAX=0.02,DSMIN=0.002,PARMIN=0,PARMAX=25
@ AUTOXMIN=0,AUTOXMAX=25,AUTOYMIN=0,AUTOYMAX=50
done
```

To calculate bifurcation diagrams (Figs 2I, 4A, S3A-C and S3I, K), dynamic variable of Sub_T in the code has to be made as a constant parameter with a value of 50 nM, in order to have a steady state for the probe.

The model with Staurosporine effect

Staurosporine is a competitive inhibitor of ATP binding to protein-kinases, therefore it blocks the activity of both Cdk and Gwl. In our model, Staurosporine binds with the same affinity to both Cdk and all three forms of Gwl kinase, thereby inhibiting their kinase activities including auto-phosphorylation of Gwl (Fig. S4E). The model is applicable at low Staurosporine concentration as well because it does not assume constant free inhibitor concentration. The following XPPAut code can be used to simulate Staurosporine effect on Figs. S4A,B.

```
# .ode file for time-course simulation of the Staurosporine effect
dSubT/dt = - kdSub*SubT
dpSub/dt =  kCdkSub*Cdka*(SubT-pSub) - (kB55Sub*B55 +  kdSub)*pSub
dCdka/dt = kdisSts*(Cdk - Cdka) - kasSts*Cdka*(10^logStau - (Cdk - Cdka) - (Gwltot - Gwlfree))
dpENSA/dt = kGwENSA*pGwlp*ENSA + kdiss*B55pENSA - kass*pENSA*B55 - kCdkENSA*Cdka*pENSA
+ kcatB55T*pENSApB55
dB55pENSA/dt = kass*pENSA*B55 - kdiss*B55pENSA - kcatB55*B55pENSA
dpENSAp/dt = kCdkENSA*Cdka*pENSA + kdissT*pENSApB55 - kassT*pENSAp*B55 +
kGwENSA*pGwlp*ENSAp + kdiss*B55pENSAp - kass*B55*pENSAp
dpENSApB55/dt = kassT*pENSAp*B55 - kdissT*pENSApB55 - kcatB55T*pENSApB55
dB55pENSAp/dt = kass*pENSAp*B55 - kdiss*B55pENSAp - kcatB55*B55pENSAp
dENSAp/dt = kcatB55*B55pENSAp - kassT*ENSAp*B55 + kdissT*ENSApB55 - kGwENSA*pGwlp*ENSAp
+ kCdkENSA*Cdka*ENSA
dENSApB55/dt = kassT*B55*ENSAp - kdissT*ENSApB55 - kcatB55T*ENSApB55
dGwlt/dt =  kCdkGw*Cdka*(Gwltot - Gwlt) - kB55Gw*B55*alfa*B55*Gwlt/(alfa*B55 + Gwlfree/Gwltot)
dGwlfree/dt = kdisSts*(Gwltot - Gwlfree) - kasSts*Gwlfree*(10^logStau - (Cdk - Cdka) - (Gwltot - Gwlfree))
pGwlt = alfa*B55*Gwlt/(alfa*B55 + Gwlfree/Gwltot)
pGwlp = Gwlfree*(Gwlt - pGwlt)/Gwltot
B55 = B55T - B55pENSA - pENSApB55 - B55pENSAp - ENSApB55
ENSA = ENSAtot - pENSA - B55pENSA - pENSAp - pENSApB55 - B55pENSAp - ENSAp - ENSApB55
aux S67pENSA = pENSA + B55pENSA + pENSAp + B55pENSAp + pENSApB55
aux pGwlp = pGwlt/(1 + alfa*B55)
aux B55 = B55T - B55pENSA - pENSApB55 - B55pENSAp - ENSApB55
init SubT=50, pSub=0, Cdka=20, Gwlfree=20

par logStau=-10, Cdk=20, kdisSts=0.015, kasSts=0.0001
par kCdkSub=0.0073, kB55Sub=0.0013, kdSub=0.002
par ENSAtot=300, B55T=50, kGwENSA=0.2358
par kass=0.3350, kdiss=0.0267, kcatB55=2.7504
par Gwltot=20, kCdkGw=0.0012, kB55Gw=0.1447, alfa=0.003
par kCdkENSA=0.0016, kassT=1.6068, kdissT=145.256, kcatB55T=0.5211

@ total=25,dt=1,meth=STIFF,xp=time,yp=pSub,xlo=0,xhi=60,ylo=0,yhi=50
@ NTST=50,NMAX=100000000,NPR=1000000,DS=0.01,BOUNDS=2000
@ DSMAX=0.01,DSMIN=0.001,PARMIN=0,PARMAX=3.5
@ AUTOXMIN=1,AUTOXMAX=2.5,AUTOYMIN=0,AUTOYMAX=50
done
```

To calculate bifurcation diagrams (Fig. 3C, Fig. S4F), dynamic variable of Sub_T in the code has to be made as a constant parameter with a value of 50 nM, in order to have a steady state for the probe.

Estimation of the kinetic parameters of the model

Although the concentrations within the reconstituted system are known, the model also contains numerous kinetic parameters whose values have to be estimated. $k_{Cdk,Sub}$ was determined by phosphorylation of the probe with different concentrations of Cdk2:CycA complex in the absence of PP2A:B55, in which case the phosphorylated (pSub) probe follows the time course:

$$[pSub] = [Sub]_T \cdot \left(1 - e^{-k_{Cdk,Sub} \cdot [Cdk] \cdot t}\right) \tag{9}$$

The Cdk dependence of the specific phosphorylation rate:

$$\frac{1}{[Sub]} \frac{d[Sub]}{dt} = -k_{Cdk,Sub} \cdot [Cdk] \tag{10}$$

provides $k_{Cdk,Sub}$. In the presence of PP2A:B55, the probe phosphorylation rapidly reaches a steady state ($pSub_{ss}$), whose value is hyperbolically dependent on Cdk concentration:

$$[pSub]_{ss} = \frac{[Sub]_T \cdot k_{Cdk,Sub} \cdot [Cdk]}{k_{B55,Sub} \cdot [B55] + k_{Cdk,Sub} \cdot [Cdk]} \tag{11}$$

which can be linearized similarly to the Lineweaver-Burk plot of the Michaelis-Menten equation:

$$\frac{1}{[pSub]_{ss}} = \frac{1}{[Sub]_T} + \frac{k_{B55,Sub} \cdot [B55]}{[Sub]_T \cdot k_{Cdk,Sub}} \cdot \frac{1}{[Cdk]} \tag{12}$$

and the slope of the line provides the value of $k_{B55,Sub}$. An alternative estimate of $k_{B55,Sub}$ is provided by the exponential decay of the phosphorylated probe after p27 addition.

The kinetic parameters of ENSA and PP2A:B55 interactions were already characterized [S10] with $K_M \cong 1$ nM and $k_{cat} = 3$ min^{-1}. These values were used as initial guesses in the search of the values of the remaining parameters using Matlab routine MEIGO [S11]. The kinetic parameters of the model are summarized in Table S1.

Estimation of the concentration of free PP2A:B55 from the rate of dephosphorylation of the probe

Figs. S4C, S4G illustrate the release of PP2A:B55 from the complex with pENSA after p27 inhibition of Cdk or Staurosporine inhibition of both protein kinases. Assuming complete block of probe phosphorylation by high concentration of kinase inhibitors, the specific rate of change for phosphorylated probe (pSub) is given:

$$\frac{1}{[pSub]} \frac{d[pSub]}{dt} = -k_{B55,Sub} \cdot [B55]$$

The specific rate of change of [pSub] was estimated by fitting a second-order polynomial to odd number (N=15, 17 or 19) experimental values of pSub (in nM) using the method of least squares and dividing the first derivative of the fitted curve by $[pSub]$ at the central point. This procedure was repeated for successive central points and dividing the specific rates by the constant value of $k_{B55,Sub}$ provided the B55 time-course.

Supplemental References

S1. Saito, K., Chang, Y.F., Horikawa, K., Hatsugai, N., Higuchi, Y., Hashida, M., Yoshida, Y., Matsuda, T., Arai, Y., and Nagai, T. (2012). Luminescent proteins for high-speed single-cell and whole-body imaging. Nat Commun *3*, 1262.

S2. Blake-Hodek, K.A., Williams, B.C., Zhao, Y., Castilho, P.V., Chen, W., Mao, Y., Yamamoto, T.M., and Goldberg, M.L. (2012). Determinants for activation of the atypical AGC kinase Greatwall during M phase entry. Molecular and cellular biology *32*, 1337-1353.

S3. Murray, A.W., and Kirschner, M.W. (1989). Cyclin synthesis drives the early embryonic cell cycle. Nature *339*, 275-280.

S4. Mochida, S., Ikeo, S., Gannon, J., and Hunt, T. (2009). Regulated activity of PP2A-B55 delta is crucial for controlling entry into and exit from mitosis in Xenopus egg extracts. Embo J *28*, 2777-2785.

S5. Mochida, S., Maslen, S.L., Skehel, M., and Hunt, T. (2010). Greatwall phosphorylates an inhibitor of protein phosphatase 2A that is essential for mitosis. Science *330*, 1670-1673.

S6. Mochida, S. (2014). Regulation of alpha-endosulfine, an inhibitor of protein phosphatase 2A, by multisite phosphorylation. FEBS J *281*, 1159-1169.

S7. Castilho, P.V., Williams, B.C., Mochida, S., Zhao, Y., and Goldberg, M.L. (2009). The M phase kinase Greatwall (Gwl) promotes inactivation of PP2A/B55delta, a phosphatase directed against CDK phosphosites. Mol Biol Cell *20*, 4777-4789.

S8. Brown, N.R., Noble, M.E., Endicott, J.A., Garman, E.F., Wakatsuki, S., Mitchell, E., Rasmussen, B., Hunt, T., and Johnson, L.N. (1995). The crystal structure of cyclin A. Structure *3*, 1235-1247.

S9. Kusubata, M., Tokui, T., Matsuoka, Y., Okumura, E., Tachibana, K., Hisanaga, S., Kishimoto, T., Yasuda, H., Kamijo, M., Ohba, Y., et al. (1992). p13suc1 suppresses the catalytic function of p34cdc2 kinase for intermediate filament proteins, in vitro. J Biol Chem *267*, 20937-20942.

S10. Williams, B.C., Filter, J.J., Blake-Hodek, K.A., Wadzinski, B.E., Fuda, N.J., Shalloway, D., and Goldberg, M.L. (2014). Greatwall-phosphorylated Endosulfine is both an inhibitor and a substrate of PP2A-B55 heterotrimers. eLife *3*, e01695.

S11. Egea, J.A., Henriques, D., Cokelaer, T., Villaverde, A.F., MacNamara, A., Danciu, D.P., Banga, J.R., and Saez-Rodriguez, J. (2014). MEIGO: an open-source software suite based on metaheuristics for global optimization in systems biology and bioinformatics. BMC Bioinformatics *15*, 136.
