# Supplementary material for: Two Bistable Switches Govern M Phase Entry
Source: Curr Biol. 2016 Dec 19;26(24):3361–7. doi: 10.1016/j.cub.2016.10.022 (PMC5196020; doi:10.1016/j.cub.2016.10.022)
Supplement: Document S2. Article plus Supplemental Information [file mmc2.pdf]

Current Biology

Report

Two Bistable Switches Govern M Phase Entry

Graphical Abstract

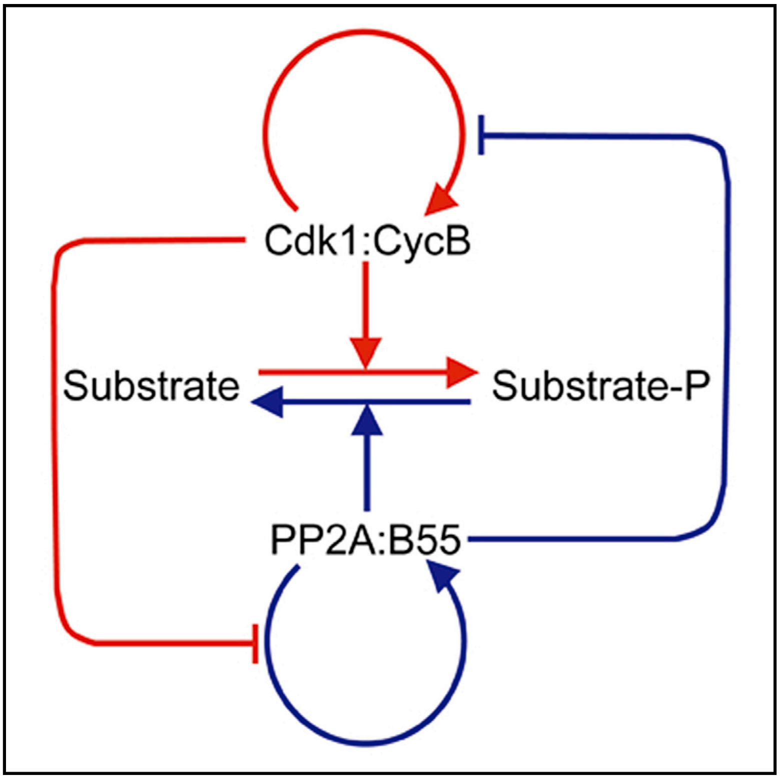

Highlights

- Cdk1 auto-activation loop is dispensable for switch-like mitotic entry
- PP2A:B55 auto-regulation creates a bistable switch
- Two bistable switches provide a robust solution for mitotic entry

Authors

Satoru Mochida, Scott Rata, Hirotsugu Hino, Takeharu Nagai, Béla Novák

Correspondence

mochida@kumamoto-u.ac.jp (S.M.), bela.novak@bioch.ox.ac.uk (B.N.)

In Brief

Mochida et al. find, using a biochemical reconstitution and mathematical simulation, that the regulation of PP2A:B55 phosphatase can create a bistable switch for mitotic phosphorylation of Cdk1 substrates. This new mechanism, together with the Cdk1 auto-activation loop, provides a robust solution for irreversible and switch-like mitotic entry.

Mochida et al., 2016, Current Biology *26*, 3361–3367
December 19, 2016 © 2016 Elsevier Ltd.
http://dx.doi.org/10.1016/j.cub.2016.10.022

Current Biology
Report

Two Bistable Switches Govern M Phase Entry

Satoru Mochida,[1,2,3,4,8,*] Scott Rata,[5] Hirotsugu Hino,[1,7] Takeharu Nagai,[6] and Béla Novák[5,*]
[1]Priority Organization for Innovation and Excellence
[2]Institute for Medical Embryology and Genetics
[3]International Research Center for Medical Science
Kumamoto University, Kyoyoto-honjo 1, 2-2-1 Honjo, Chuo-ku, Kumamoto 860-0811, Japan
[4]Precursory Research for Embryonic Science and Technology (PRESTO) Program, Japan Science and Technology Agency, 4-1-8 Honcho, Kawaguchi, Saitama 332-0012, Japan
[5]Department of Biochemistry, University of Oxford, South Parks Road, Oxford OX1 3QU, UK
[6]The Institute of Scientific and Industrial Research, Osaka University, Mihogaoka 8-1, Ibaraki, Osaka 567-0047, Japan
[7]Present address: Department of Biochemistry, Tokyo Medical University, 6-1-1 Shinjuku, Shinjuku-ku, Tokyo 160-8402, Japan
[8]Lead Contact
*Correspondence: mochida@kumamoto-u.ac.jp (S.M.), bela.novak@bioch.ox.ac.uk (B.N.)
http://dx.doi.org/10.1016/j.cub.2016.10.022

SUMMARY

The abrupt and irreversible transition from interphase to M phase is essential to separate DNA replication from chromosome segregation. This transition requires the switch-like phosphorylation of hundreds of proteins by the cyclin-dependent kinase 1 (Cdk1):cyclin B (CycB) complex. Previous studies have ascribed these switch-like phosphorylations to the auto-activation of Cdk1:CycB through the removal of inhibitory phosphorylations on Cdk1-Tyr15 [1, 2]. The positive feedback in Cdk1 activation creates a bistable switch that makes mitotic commitment irreversible [2–4]. Here, we surprisingly find that Cdk1 auto-activation is dispensable for irreversible, switch-like mitotic entry due to a second mechanism, whereby Cdk1:CycB inhibits its counteracting phosphatase (PP2A:B55). We show that the PP2A:B55-inhibiting Greatwall (Gwl)-endosulfine (ENSA) pathway is both necessary and sufficient for switch-like phosphorylations of mitotic substrates. Using purified components of the Gwl-ENSA pathway in a reconstituted system, we found a sharp Cdk1 threshold for phosphorylation of a luminescent mitotic substrate. The Cdk1 threshold to induce mitotic phosphorylation is distinctly higher than the Cdk1 threshold required to maintain these phosphorylations—evidence for bistability. A combination of mathematical modeling and biochemical reconstitution show that the bistable behavior of the Gwl-ENSA pathway emerges from its mutual antagonism with PP2A:B55. Our results demonstrate that two interlinked bistable mechanisms provide a robust solution for irreversible and switch-like mitotic entry.

RESULTS AND DISCUSSION

Cell-cycle progression requires rapid and irreversible cellular decisions at the transitions between phases. A key transition is the entry into mitosis, which requires the phosphorylation of hundreds of proteins by cyclin-dependent kinase 1 (Cdk1):cyclin B (CycB) to bring about processes such as nuclear envelope breakdown, chromosome condensation, and spindle assembly. The basic requirements of rapid and irreversible transitions are met, because the response to CycB is governed by a fast, bistable switch [2–4]. Below a CycB threshold, the cell remains in interphase, because Cdk1:CycB complexes are inactivated by phosphorylation at Cdk1-Tyr15 (Y15). Above the CycB threshold, the inhibitory Y15 phosphorylation is removed, because the Y15 kinases (Wee1/Myt1) are turned off while the Y15 phosphatase (Cdc25) is turned on [1]. Activated Cdk1 phosphorylates many mitotic substrates, including Wee1/Myt1 and Cdc25; phosphorylating these Y15-modifying enzymes makes the system auto-catalytic. However, Cdk1 also performs phosphorylations that inhibit the counteracting phosphatase PP2A:B55 that eventually removes mitotic phosphorylations at M phase exit and in interphase [5–7]. This led us to ask whether regulation of PP2A:B55 plays a role in creating the Cdk activity threshold for mitotic substrate phosphorylations.

We induced phosphorylation and dephosphorylation of mitotic proteins in *Xenopus* egg extracts by the addition of either non-degradable CycB (CycB-ΔN) or the Cdk inhibitor protein $p27^{Kip1}$ (Figure 1A). To quantitatively analyze the ratio of Cdk and PP2A:B55 activities, we developed a set of luminescent probes whose light emittance reflects their phosphorylation level (Figures S1A–S1C; see also the Supplemental Experimental Procedures). The phosphorylation and light emittance of our probes correlated well with the phosphorylation/dephosphorylation cycle of mitotic proteins (Figures 1A, 1B, and S1D). We also confirmed that mitotic phosphorylations required CycB levels to be higher than a threshold (Figure 1C, Mock). At low CycB levels (at and below 108 nM), Cdk1 becomes Y15 phosphorylated (an inactive state), and both the probe and the endogenous proteins remained dephosphorylated (Figures 1C and 1D, Mock). Above a critical CycB threshold (126 nM), Cdk1 became activated by the dephosphorylation of Y15, and mitotic phosphorylations were detected (Figures 1C and 1D, Mock). We conclude that the CycB threshold of Cdk1 activation coincides with mitotic phosphorylation at the G2/M transition and that our probes can detect this threshold in *Xenopus* egg extracts.

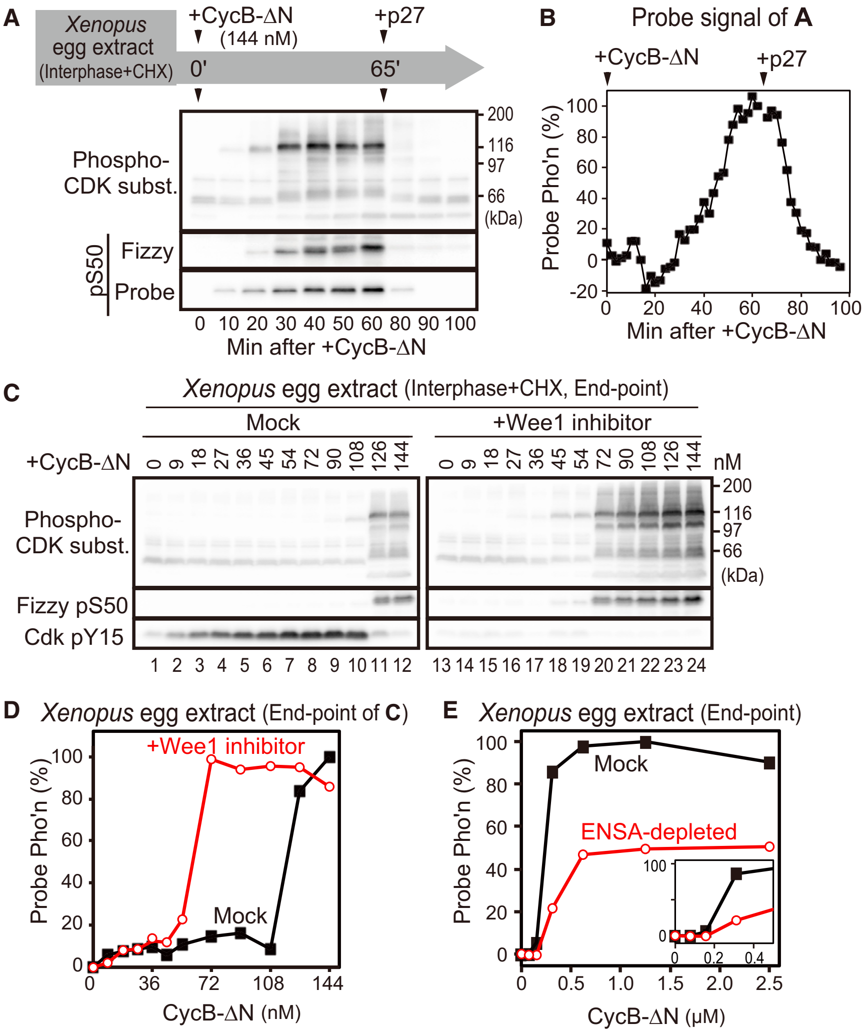

Figure 1. CycB Threshold of Mitotic Phosphorylation in *Xenopus* Egg Extracts Persists in the Presence of an Inhibitor of Wee1

(A) Interphase egg extract was supplemented with luminescent probe, CycB-ΔN (144 nM) and cycloheximide (CHX) at 0 min. $p27^{Kip1}$ (450 nM) was added at 65 min for mitotic exit. Aliquots were analyzed by immunoblotting for phosphorylation (Pho'n) of CDK substrates (subst.), endogenous Fizzy-Ser50, and the probe. The probe was only weakly phosphorylated when CycB-ΔN was not added (Figure S1D).

(B) Probe luminescence of (A) was plotted.

(C) Interphase egg extract was supplemented with increasing amount of CycB-ΔN in the absence (left) or presence (right) of PD166285 (1 μM). After 80 min incubation, aliquots were analyzed by immunoblotting.

(D) Probe luminescence of the samples shown in (C) was plotted.

(E) Interphase egg extract immunodepleted of ENSA was analyzed as in (D). The inset is a magnification of the low x-axis range. Experiments were repeated three to five times, and similar results were obtained.

The S50-1G4 probe was used in this figure. See also Figure S1.

It is well established that the CycB threshold of *Cdk1 activation* due to Y15 phosphorylation [1, 2] can be abolished by the inhibition of Wee1/Myt1 kinases [8]. Surprisingly, however, a sharp CycB threshold of *mitotic phosphorylation* persisted at a lower level of CycB (about 60 nM) when the Wee1/Myt1 inhibitor PD166285 was added to the extract, despite the lack of Y15 phosphorylation (Figures 1C and 1D). Therefore, we must distinguish two related, but not identical, thresholds: a threshold of Cdk1 activation and that of mitotic phosphorylation. Since Cdk1 activity is directly proportional to CycB levels in the absence of Cdk1-Y15 phosphorylation [8], Figures 1C and 1D indicate a Cdk1 activity threshold of mitotic phosphorylation that exists independently of the Cdk1 auto-activation mechanism.

One way to establish a Y15-independent threshold of mitotic phosphorylation would be switch-like inactivation of a Cdk1-countering phosphatase, one obvious candidate being PP2A:B55. This enzyme is inhibited by the Greatwall-kinase (Gwl)-phosphorylated form of alpha-endosulfine (ENSA) [6, 7, 9–11]. Because Gwl is activated by Cdk1 phosphorylation, Cdk1 inhibits PP2A:B55 via this Gwl-ENSA pathway, allowing the high phosphorylation level of Cdk1 substrates in mitosis [5, 12]. Therefore, we tested whether the Gwl-ENSA pathway helps establish the CycB threshold of mitotic phosphorylation in *Xenopus* egg extract. In the absence of ENSA, probe phosphorylation level showed a sigmoidal response to CycB levels with half the plateau level of the control sample, suggesting that the Cdk1 auto-activation mechanism can still operate in the absence of PP2A:B55 inhibition (Figures 1E and S1E). We conclude that the Gwl-ENSA pathway plays a major role in full, switch-like phosphorylation of mitotic substrates in response to the levels of CycB and Cdk1 activity, even when the Cdk1 auto-activation mechanism is intact.

To test whether the Gwl-ENSA pathway, together with Cdk1:CycB and PP2A:B55, can create a threshold by themselves in the absence of Cdk1 auto-activation, we next developed a reconstituted system of the Gwl-ENSA pathway using purified proteins (Figure S2A). As would be expected, in the absence of ENSA, the phosphorylation of both the probe and Gwl showed a graded response to Cdk1 activity with no threshold (Figures 2A, 2B, 2E, and 2F).

In the presence of the complete Gwl-ENSA pathway in the reconstitution system, Cdk1 phosphorylation of the mitotic substrates showed a switch-like response at a Cdk1:CycB concentration between 5.1 and 6.8 nM (Figures 2C–2F). The most dramatic phosphorylation shift was shown by ENSA, which was converted from the unphosphorylated to the phosphorylated form around the Cdk threshold (Figure 2F). The band shift of ENSA is caused by Gwl-catalyzed S67 phosphorylation of ENSA, which is responsible for ENSA binding to PP2A:B55

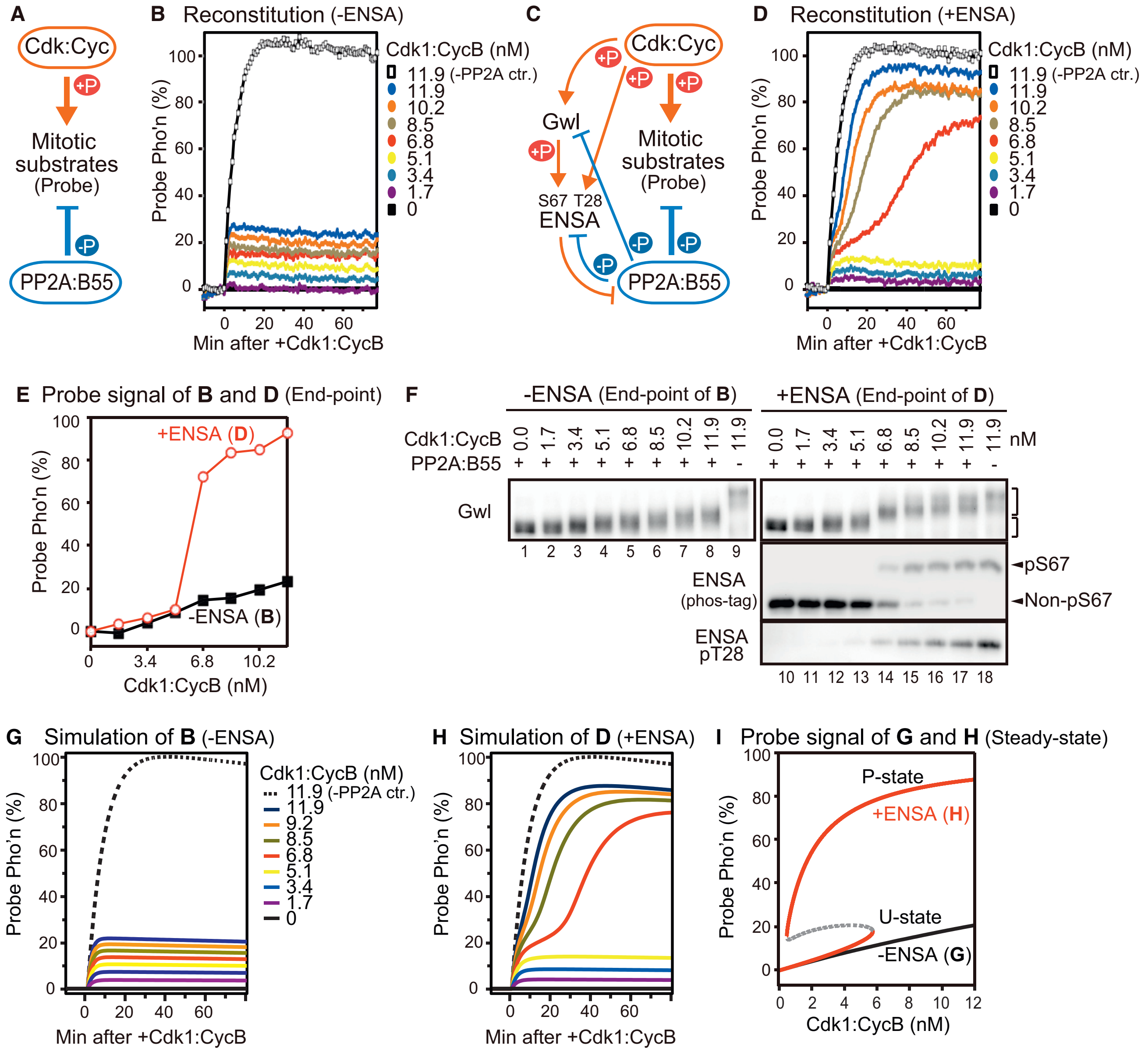

Figure 2. The Gwl-ENSA Pathway Creates a Cdk Activity Threshold of Mitotic Phosphorylation in a Reconstituted System

(A) Simple Cdk:Cyc/PP2A:B55 antagonism. Red and blue represent mitotic and anti-mitotic effects, respectively.

(B) Time-course analysis of probe phosphorylation (Pho'n) shown as in (A), with increasing Cdk1:CycB levels ([PP2A:B55] = 50 nM). A sample without PP2A:B55 was used as 100% control (ctr.; open black squares).

(C) Cdk:Cyc/PP2A:B55 antagonism with the Gwl-ENSA pathway.

(D) Time-course analysis of probe phosphorylation shown as in (C) ([PP2A:B55] = 50 nM, [Gwl] = 20 nM, and [ENSA] = 300 nM). (E) Endpoint analyses of (B) (black) and (D) (red).

(F) Aliquots of (B) and (D) were analyzed for Gwl (top), ENSA-S67 phosphorylation (middle; upper arrowhead indicates S67-phosphorylated form of ENSA), and ENSA-T28 phosphorylation (bottom). Slower and faster migrating forms of Gwl are indicated by square brackets. Samples free from PP2A:B55 are shown in lanes 9 and 18 as fully phosphorylated controls. Representative result of five experiments is shown here.

(G and H) Simulation analyses of probe phosphorylation of (B) and (D).

(I) The steady states of phosphorylated probe in (G) and (H), which can be directly compared with the endpoint analysis panel in (E).

The T50-NCP probe was used in this figure. See also Figures S2 and S3 and Tables S1 and S2.

[13]. In both the presence and absence of ENSA, both the probe and Gwl are partially phosphorylated at low-Cdk activities to a similar extent, suggesting that PP2A:B55 is not inhibited below the Cdk threshold of probe phosphorylation. The sharp increase in probe phosphorylation above the Cdk threshold coincides with ENSA S67 phosphorylation, which titrates the phosphatase away from other phosphorylated substrates (e.g., both the probe and Gwl).

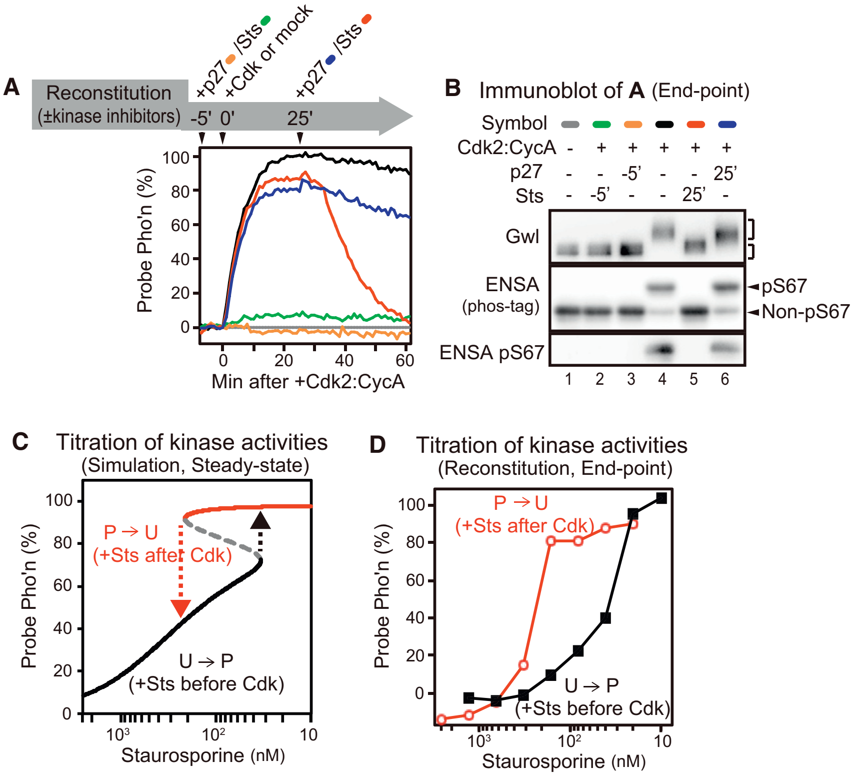

Figure 3. The Gwl-ENSA Pathway Defines a Bistable Switch with Two Distinct Thresholds

(A) Reconstituted system was supplemented either with p27^{Kip1} (450 nM) or staurosporine (Sts) (10 μM) 5 min before or 25 min after adding a supra-threshold level of Cdk activity (20 nM of Cdk2:CycA). Pho'n, phosphorylation.

(B) Endpoint analysis of the samples shown in (A) was done for Gwl and ENSA-S67 phosphorylation by immunoblotting.

(C) Simulation of Gwl/PP2A:B55 antagonism with staurosporine titration suggests bistability of the reconstitution system.

(D) Experimental confirmation of bistability suggested in (C). Different concentrations of staurosporine were added to the reconstituted system before (black) or after (red) the addition of a supra-threshold level of Cdk activity (20 nM of Cdk2:CycA). Data after 60 min incubation were plotted. Representative data of two experiments are shown. The S50-1G12 probe was used in this figure. See also Figures S4A–S4F and Tables S1 and S2.

The existence of a Cdk threshold of mitotic probe phosphorylation in the steady-state response of the reconstituted system implies that all components of the system, including Gwl, participate in inactivating, as well as activating, reactions. If Gwl were not inactivated by PP2A:B55, there would be no Cdk threshold, since Gwl would eventually be fully activated at all Cdk activities. We therefore surmise that Gwl is a substrate of the PP2A:B55 phosphatase (Figure 2C), which keeps Gwl dephosphorylated at subthreshold Cdk activities. Furthermore, inactivation of PP2A:B55 by S67-phosphorylated ENSA (pENSA) causes a switch-like phosphorylation of Gwl above the Cdk threshold. This effect creates an antagonistic relationship between Gwl and PP2A:B55, which can produce a Cdk1 threshold for the mitotic phosphorylation [14, 15].

To better understand this antagonistic relationship, we formulated mathematical models of the Gwl-ENSA pathway (Supplemental Information). At the heart of the model is the double-negative feedback between Gwl and PP2A:B55 (Figures 2C and S2B): Gwl inhibits PP2A:B55 by activation of ENSA through S67 phosphorylation, and PP2A:B55 keeps Gwl inactive by dephosphorylating it. For completeness, we also include Gwl auto-phosphorylation, which takes place after Cdk-induced priming phosphorylation [5]. The model also contains the "unfair competition" mechanism of pENSA on the phosphatase [11]: the strong binding of pENSA to PP2A:B55 titrates the phosphatase away from all other substrates and makes itself the preferential, albeit sluggish, substrate.

The mathematical model explains the sharp transition of the Gwl-ENSA pathway from an un- or hypo-phosphorylated "U" state to a hyper-phosphorylated "P" state by a bistable switch operating at the Cdk threshold of mitotic probe phosphorylation (Figures 2G–2I and S3A–S3C). This bistable switch makes the strong and experimentally testable prediction that the system will display hysteresis. At first, the Cdk threshold for turning on the Gwl-ENSA pathway at the U→P transition (the ON threshold) must be larger than the threshold for turning it off at the P→U transition (the OFF threshold).

We used Cdk2:CycA in the hysteresis experiments as the source of Cdk activity, because Cdk1:CycB is difficult to obtain in reasonable amounts from *Xenopus* egg extract or from insect cells. Cdk2:CycA behaves identically to Cdk1:CycB in all features in the reconstituted system, especially in producing the same Cdk threshold of mitotic probe phosphorylation (Figures S3D–S3K). p27^{Kip1} was added to the reconstituted system in the "U" and in the "P" states. Starting from the "U" state, p27^{Kip1} efficiently blocked phosphorylation of Gwl, ENSA, and the probe in the presence of a supra-threshold level (20 nM) of Cdk2:CycA (Figures 3A and 3B, orange sample). However, when p27^{Kip1} was added to the "P" state, it was insufficient to induce dephosphorylation of any of these proteins within the experimental time course (Figures 3A and 3B, blue sample). This apparent irreversibility of the Gwl-ENSA pathway in the reconstituted system is already a strong indication for a very small (close to zero) Cdk threshold, because slowing down near a threshold is a signature of bistable systems. This slow reactivation of PP2A:B55 is consistent with our model, which suggests that P→U transition requires ~300 min after Cdk inhibition by p27^{Kip1} (Figures S4A and S4B). This long time delay of PP2A:B55 activation is caused by continued action of Gwl after Cdk inhibition by p27^{Kip1} (Figure 3B). Dephosphorylation of mitotic substrates also becomes delayed after p27^{Kip1}-induced mitotic exit of *Xenopus* egg extract when the activity of PP1 is inhibited (Figure S4D), which is normally responsible for the initial inactivation of Gwl [16].

Since this increased time delay makes the estimation of the OFF Cdk threshold difficult in the reconstituted system, we

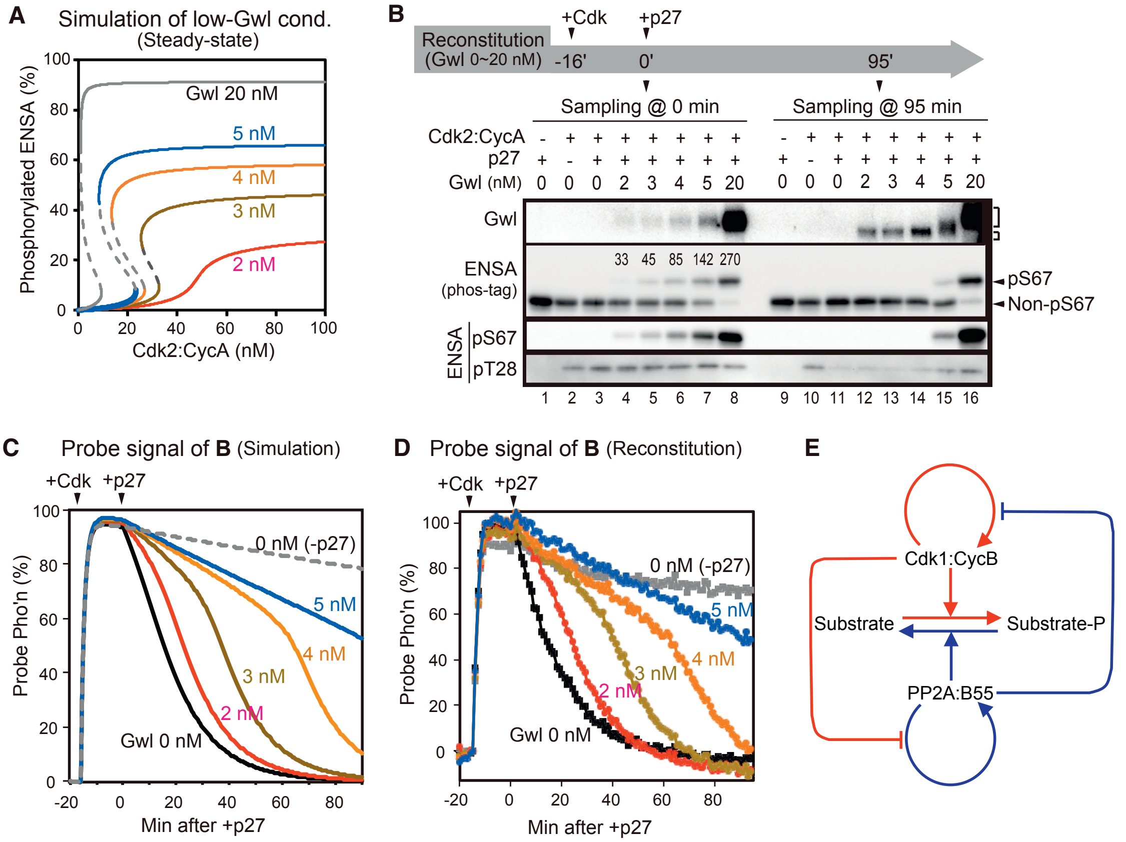

Figure 4. The Effect of Gwl Concentration on Cdk Thresholds and the Length of Time Delay before PP2A:B55 Reactivation

(A) Steady states of the pS67 form of ENSA predicted from the model for different Gwl concentrations. cond., condition.

(B) Model in (A) was experimentally tested in the reconstituted system. Samples of lower Gwl (0–20 nM) and higher Cdk (100 nM of Cdk2:CycA) concentrations were incubated for 16 min. Aliquots of samples were taken before (0 min, left) and 95 min after (right) the addition of p27^{Kip1}. Numbers in the ENSA blot (phos-tag, second from top) indicate concentrations of S67-phosphorylated ENSA in nanomolar (nM). PP2A:B55 is 50 nM. The effect of ENSA T28 phosphorylation is described in Figure S4 and the Supplemental Information.

(C and D) Simulation (C) and experimental (D) analyses of the time course of probe phosphorylation (Pho'n) shown in (B) were done. Representative data of four experiments are shown.

(E) Schematic diagram of two interlinked bistable switches.

The S50-1G12 probe was used in this figure, including the model. See also Figures S4G and S4H and Tables S1 and S2.

decided to use a generic kinase inhibitor, staurosporine, which inhibits both Cdk and Gwl activities. 10 μM staurosporine added to the reconstituted system in the "P" state induced dephosphorylation of all proteins (Figure 3B, red sample). However, this staurosporine-induced probe dephosphorylation was also characterized by a well-defined short time delay (5 min) (Figure 3A, red line, 25–30 min). This short time delay would be the consequence of "unfair competition" between pENSA and the probe for PP2A:B55 [11, 17], which is terminated by pENSA dephosphorylation by PP2A:B55 (Figure S4C, red curve). The "unfair competition" is drastically lengthened after p27^{Kip1}-induced Cdk inhibition by the mutual antagonism between Gwl and PP2A:B55 (Figures S4B and S4C, blue curves).

The mathematical model also predicts different ON and OFF thresholds when both Cdk and Gwl are inhibited by staurosporine (Figures 3C, S4E, and S4F). The hysteresis effect with staurosporine is caused by synergetic Gwl-inhibitory effects of the inhibitor and PP2A:B55 rather than different sensitivities of the two kinases to staurosporine. To test this prediction of different ON and OFF thresholds, we added exponentially increasing concentrations of staurosporine to the reconstituted system before and 25 min after the addition of 20 nM (suprathreshold) Cdk2:CycA. We then measured the steady-state level (endpoint) of probe phosphorylation (Figure 3D). 40 nM staurosporine started to suppress probe phosphorylation at the U→P transition, while 320 nM staurosporine was required to induce dephosphorylation at the P→U transition. From this result, we conclude that the reconstituted Gwl-ENSA pathway represents a bistable switch with two distinct thresholds for kinase activities.

We next asked whether we could obtain further evidence for the hysteresis of the reconstituted Gwl-ENSA pathway by manipulating the concentrations of Gwl and Cdk to decrease the time delay after the addition of p27^{Kip1}. Our model predicts that both the ON and OFF Cdk thresholds of mitotic phosphorylation are increased with the lowering of Gwl concentration (Figure 4A), and an increase of the OFF threshold provides an opportunity to decrease the time delay after Cdk inhibition by

p27^{Kip1}. Intuitively, at low-Gwl concentration, more Cdk is required to turn on the system (U→P), but it switches off faster after Cdk inhibition (P→U).

To test this prediction, we induced the "P state" at low Gwl levels using a supra-threshold concentration (100 nM) of Cdk2:CycA in the reconstitution system for 16 min before adding p27^{Kip1} (time = 0 in Figure 4B). S67 phosphorylation of ENSA just before p27^{Kip1} addition was increased with increasing Gwl levels, reaching stoichiometric balance with PP2A:B55 around 3 nM Gwl concentrations (Figure 4B, lanes 4–8). As we expected, phosphorylated ENSA and phosphorylated Gwl disappeared 95 min after the addition of p27^{Kip1} in the samples containing Gwl at 2–4 nM, an indication of P→U transition (Figure 4B, lanes 12–14). To get more insights about this experiment, we analyzed probe dephosphorylation of the same set of samples. As expected from the model, increasing Gwl concentrations delayed probe dephosphorylation in the reconstituted system, with a strikingly biphasic response at higher Gwl concentrations (Figures 4C and 4D). Assuming that Cdk inhibition is complete after p27^{Kip1} addition, the curves of Figure 4D allowed us to calculate the time course of release of PP2A:B55 from ENSA (Figure S4G; see the Supplemental Information). These calculations show a relatively abrupt activation of PP2A:B55 from pENSA inhibition, which makes the dephosphorylation of the probe biphasic. Since the model prediction matched well with the experiment (Figure S4H), this result further supports the idea that the reconstituted pathway has hysteresis.

Our work identifies the Gwl-ENSA-PP2A:B55 pathway as a bistable switch in the reconstituted system and also as a source of the Cdk1 threshold of mitotic phosphorylation in *Xenopus* egg extracts. The present work, together with the existence of the Cdk1 auto-activation loop through Y15 phosphorylation, led us to conclude that the eukaryotic mitotic control system is equipped with two bistable switches (Figure 4E). These two systems mutually inhibit each other to ensure that the two switches occupy opposite states (Cdk1 ON, PP2A:B55 OFF and vice versa). This mutual regulation is dictated by PP2A:B55 dephosphorylation of Cdk1-Y15-modifying enzymes (Wee1 and Cdc25 [18]) and by Cdk1 phosphorylation of Gwl [5, 19]. The two interlinked switches create a robust solution for switch-like mitotic substrate phosphorylation. This robustness is underlined by our results showing that the Gwl-ENSA-PP2A:B55 pathway can by itself maintain switch-like mitotic entry, even when Cdk1-Y15 phosphorylation is compromised; a similar situation can also be observed in proliferating fission yeast [20].

Having two interlinked bistable switches regulating the transitions between interphase and mitosis enhances the difference between the ON and OFF Cdk1 thresholds, which makes state transitions more challenging. Therefore, additional components ("triggers") might facilitate the transitions of the switch system from one stable state to the other. CycA-dependent kinases have been suggested as triggers for mitotic entry, as they have considerable kinase activity in interphase [21, 22], consistent with our observation that Cdk2:CycA can activate the Gwl-ENSA pathway. On mitotic exit, in contrast, dephosphorylation of Gwl by PP1 [16, 23, 24] could initiate the reverse transition by supporting the PP2A:B55 auto-activation. Whether the design principle of interlinked toggle switches with a pair of triggers is a generic feature of other decision-making pathways in living cells is a question for future experimental and modeling studies.

SUPPLEMENTAL INFORMATION

Supplemental Information includes Supplemental Experimental Procedures, four figures, and two tables and can be found with this article online at http://dx.doi.org/10.1016/j.cub.2016.10.022.

AUTHOR CONTRIBUTIONS

S.M. and B.N. designed the experiments, which S.M. performed. B.N. and S.R. performed mathematical analyses. S.M., H.H., and T.N. developed luminescent probes. B.N., S.M., and S.R. wrote the manuscript.

ACKNOWLEDGMENTS

We thank Drs. M. Bollen, N. Nakajo, Y. Shi, and J. Gannon for sharing their reagents; and Drs. T. Hunt, F. Barr, M. Goldberg, J. Tyson, and A. Murray for their advice. S.M. also thanks all lab members and the IMEG, RIC, and GTC facilities of Kumamoto University for their support. B.N. is supported by a BBSRC Strategic LoLa grant (BB/MM00354X/1). S.M. is supported by the JST-PRESTO program, JSPS-KAKENHI (24687025), and the Takeda Science Foundation. S.R. is supported by the EPSRC with a studentship (EP/G03706X/1).

Received: August 29, 2016
Revised: September 27, 2016
Accepted: October 12, 2016
Published: November 23, 2016

REFERENCES

1. Solomon, M.J., Glotzer, M., Lee, T.H., Philippe, M., and Kirschner, M.W. (1990). Cyclin activation of p34cdc2. Cell *63*, 1013–1024.
2. Novak, B., and Tyson, J.J. (1993). Numerical analysis of a comprehensive model of M-phase control in Xenopus oocyte extracts and intact embryos. J. Cell Sci. *106*, 1153–1168.
3. Sha, W., Moore, J., Chen, K., Lassaletta, A.D., Yi, C.S., Tyson, J.J., and Sible, J.C. (2003). Hysteresis drives cell-cycle transitions in Xenopus laevis egg extracts. Proc. Natl. Acad. Sci. USA *100*, 975–980.
4. Pomerening, J.R., Sontag, E.D., and Ferrell, J.E., Jr. (2003). Building a cell cycle oscillator: hysteresis and bistability in the activation of Cdc2. Nat. Cell Biol. *5*, 346–351.
5. Blake-Hodek, K.A., Williams, B.C., Zhao, Y., Castilho, P.V., Chen, W., Mao, Y., Yamamoto, T.M., and Goldberg, M.L. (2012). Determinants for activation of the atypical AGC kinase Greatwall during M phase entry. Mol. Cell. Biol. *32*, 1337–1353.
6. Gharbi-Ayachi, A., Labbé, J.C., Burgess, A., Vigneron, S., Strub, J.M., Brioudes, E., Van-Dorsselaer, A., Castro, A., and Lorca, T. (2010). The substrate of Greatwall kinase, Arpp19, controls mitosis by inhibiting protein phosphatase 2A. Science *330*, 1673–1677.
7. Mochida, S., Maslen, S.L., Skehel, M., and Hunt, T. (2010). Greatwall phosphorylates an inhibitor of protein phosphatase 2A that is essential for mitosis. Science *330*, 1670–1673.
8. Yang, Q., and Ferrell, J.E., Jr. (2013). The Cdk1-APC/C cell cycle oscillator circuit functions as a time-delayed, ultrasensitive switch. Nat. Cell Biol. *15*, 519–525.
9. Castilho, P.V., Williams, B.C., Mochida, S., Zhao, Y., and Goldberg, M.L. (2009). The M phase kinase Greatwall (Gwl) promotes inactivation of PP2A/B55delta, a phosphatase directed against CDK phosphosites. Mol. Biol. Cell *20*, 4777–4789.
10. Mochida, S., Ikeo, S., Gannon, J., and Hunt, T. (2009). Regulated activity of PP2A-B55 delta is crucial for controlling entry into and exit from mitosis in Xenopus egg extracts. EMBO J. *28*, 2777–2785.

11. Williams, B.C., Filter, J.J., Blake-Hodek, K.A., Wadzinski, B.E., Fuda, N.J., Shalloway, D., and Goldberg, M.L. (2014). Greatwall-phosphorylated Endosulfine is both an inhibitor and a substrate of PP2A-B55 heterotrimers. eLife *3*, e01695.
12. Vigneron, S., Brioudes, E., Burgess, A., Labbé, J.C., Lorca, T., and Castro, A. (2009). Greatwall maintains mitosis through regulation of PP2A. EMBO J. *28*, 2786–2793.
13. Mochida, S. (2014). Regulation of α-endosulfine, an inhibitor of protein phosphatase 2A, by multisite phosphorylation. FEBS J. *281*, 1159–1169.
14. Hégarat, N., Vesely, C., Vinod, P.K., Ocasio, C., Peter, N., Gannon, J., Oliver, A.W., Novák, B., and Hochegger, H. (2014). PP2A/B55 and Fcp1 regulate Greatwall and Ensa dephosphorylation during mitotic exit. PLoS Genet. *10*, e1004004.
15. Vinod, P.K., and Novak, B. (2015). Model scenarios for switch-like mitotic transitions. FEBS Lett. *589*, 667–671.
16. Heim, A., Konietzny, A., and Mayer, T.U. (2015). Protein phosphatase 1 is essential for Greatwall inactivation at mitotic exit. EMBO Rep. *16*, 1501–1510.
17. Cundell, M.J., Bastos, R.N., Zhang, T., Holder, J., Gruneberg, U., Novak, B., and Barr, F.A. (2013). The BEG (PP2A-B55/ENSA/Greatwall) pathway ensures cytokinesis follows chromosome separation. Mol. Cell *52*, 393–405.
18. Zhao, Y., Haccard, O., Wang, R., Yu, J., Kuang, J., Jessus, C., and Goldberg, M.L. (2008). Roles of Greatwall kinase in the regulation of cdc25 phosphatase. Mol. Biol. Cell *19*, 1317–1327.
19. Vigneron, S., Gharbi-Ayachi, A., Raymond, A.A., Burgess, A., Labbé, J.C., Labesse, G., Monsarrat, B., Lorca, T., and Castro, A. (2011). Characterization of the mechanisms controlling Greatwall activity. Mol. Cell. Biol. *31*, 2262–2275.
20. Coudreuse, D., and Nurse, P. (2010). Driving the cell cycle with a minimal CDK control network. Nature *468*, 1074–1079.
21. Fung, T.K., Ma, H.T., and Poon, R.Y. (2007). Specialized roles of the two mitotic cyclins in somatic cells: cyclin A as an activator of M phase-promoting factor. Mol. Biol. Cell *18*, 1861–1873.
22. Guadagno, T.M., and Newport, J.W. (1996). Cdk2 kinase is required for entry into mitosis as a positive regulator of Cdc2-cyclin B kinase activity. Cell *84*, 73–82.
23. Ma, S., Vigneron, S., Robert, P., Strub, J.M., Cianferani, S., Castro, A., and Lorca, T. (2016). Greatwall dephosphorylation and inactivation upon mitotic exit is triggered by PP1. J. Cell Sci. *129*, 1329–1339.
24. Rogers, S., Fey, D., McCloy, R.A., Parker, B.L., Mitchell, N.J., Payne, R.J., Daly, R.J., James, D.E., Caldon, C.E., Watkins, D.N., et al. (2016). PP1 initiates the dephosphorylation of MASTL, triggering mitotic exit and bistability in human cells. J. Cell Sci. *129*, 1340–1354.

Current Biology, Volume *26*

Supplemental Information

Two Bistable Switches Govern M Phase Entry

Satoru Mochida, Scott Rata, Hirotsugu Hino, Takeharu Nagai, and Béla Novák

Supplemental Figures

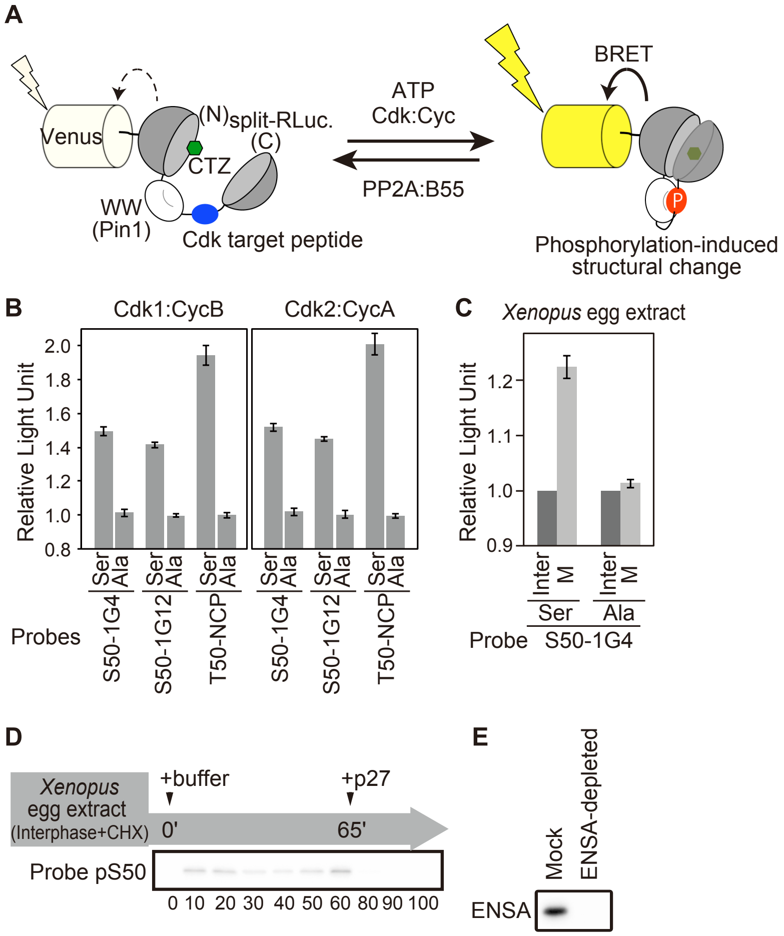

Figure S1 (Related to Figure 1): Development and analyses of the luminescent probes in purified and *Xenopus* egg extract systems. (**A**) Schematic representation of the probes [S1]. The WW domain of Pin1 and CDK-target peptide sequence were inserted into *Renilla reniformis* luciferase (RLuc). Physical interaction of WW domain with the phosphorylated peptide causes a reconstitution of the split RLuc, resulting in higher light emittance. Luciferase substrate coelenterazine-h (CTZ) is shown in green. See Methods for more details. (**B**) Probes used in this study were *in-vitro* phosphorylated by Cdk1:CycB (left) or by Cdk2:CycA (right) complexes. Luminescence 40 minutes after incubation at 30°C were plotted in relative light unit with standard deviation of three separate experiments. Probes with alanine mutation (Ala) at the phosphorylation site did not show any significant change of luminescence. (**C**) Interphase *Xenopus* egg extracts were supplemented either with S50-1G4 (Ser) or S50A-1G4 (Ala) probes. CycB-ΔN (120 nM) was added to induce mitotic entry at 23°C and luminescence after 80-minutes incubation were plotted with standard deviations of three separate experiments. (**D**) Sample without CycB-ΔN addition in Fig. 1A was analysed by immunoblotting for probe phosphorylation. (**E**) Efficiency of ENSA immunodepletion of the samples shown in Fig. 1E.

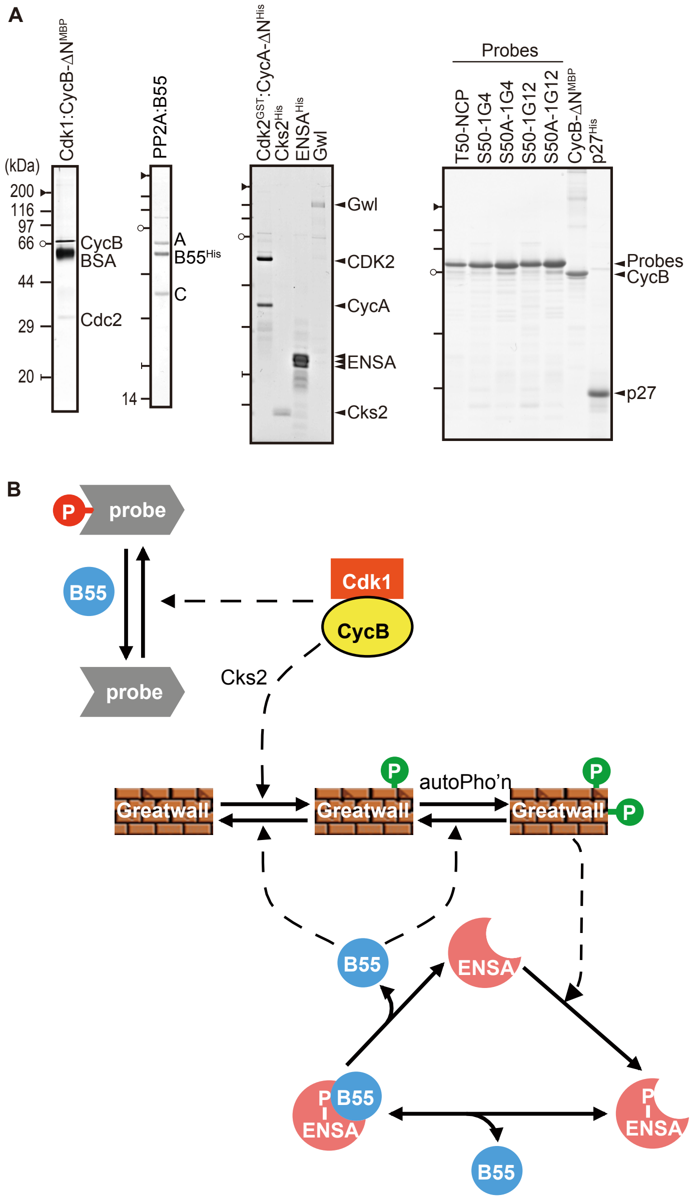

Figure S2 (Related to Figure 2): Biochemical reaction mechanism of the system reconstituted using purified proteins. (**A**) Purified protein samples separated on SDS-PAGE were either silver-stained (Cdk1:CycB) or stained with Coomassie brilliant blue. Positions of molecular weight markers are shown on the left. Arrowheads indicate positions of proteins of interest. BSA: bovine serum albumin. (**B**) This wiring diagram has been converted into differential equations of the model. Solid and dashed arrows represent chemical reactions and catalytic activities, respectively. The priming-phosphorylation of Gwl is catalysed by Cdk:cyclin complex. Cdk-activated Gwl undergoes auto-phosphorylation [S2] and the double-phosphorylated form of Gwl activates ENSA. Gwl-phosphorylated ENSA binds to PP2A:B55 and undergoes enzymatic dephosphorylation by the phosphatase. Both phosphorylations of Gwl are counter-acted by PP2A:B55 not bound to pENSA, which is the only phosphatase in our reconstituted system. Phosphorylation/dephosphorylation of the probe by Cdk:cyclin and PP2A:B55 happens downstream of the double-negative feedback between Gwl and PP2A:B55. For simplicity, T28-phosphorylation/dephosphorylation of ENSA by Cdk and PP2A:B55 are not indicated.

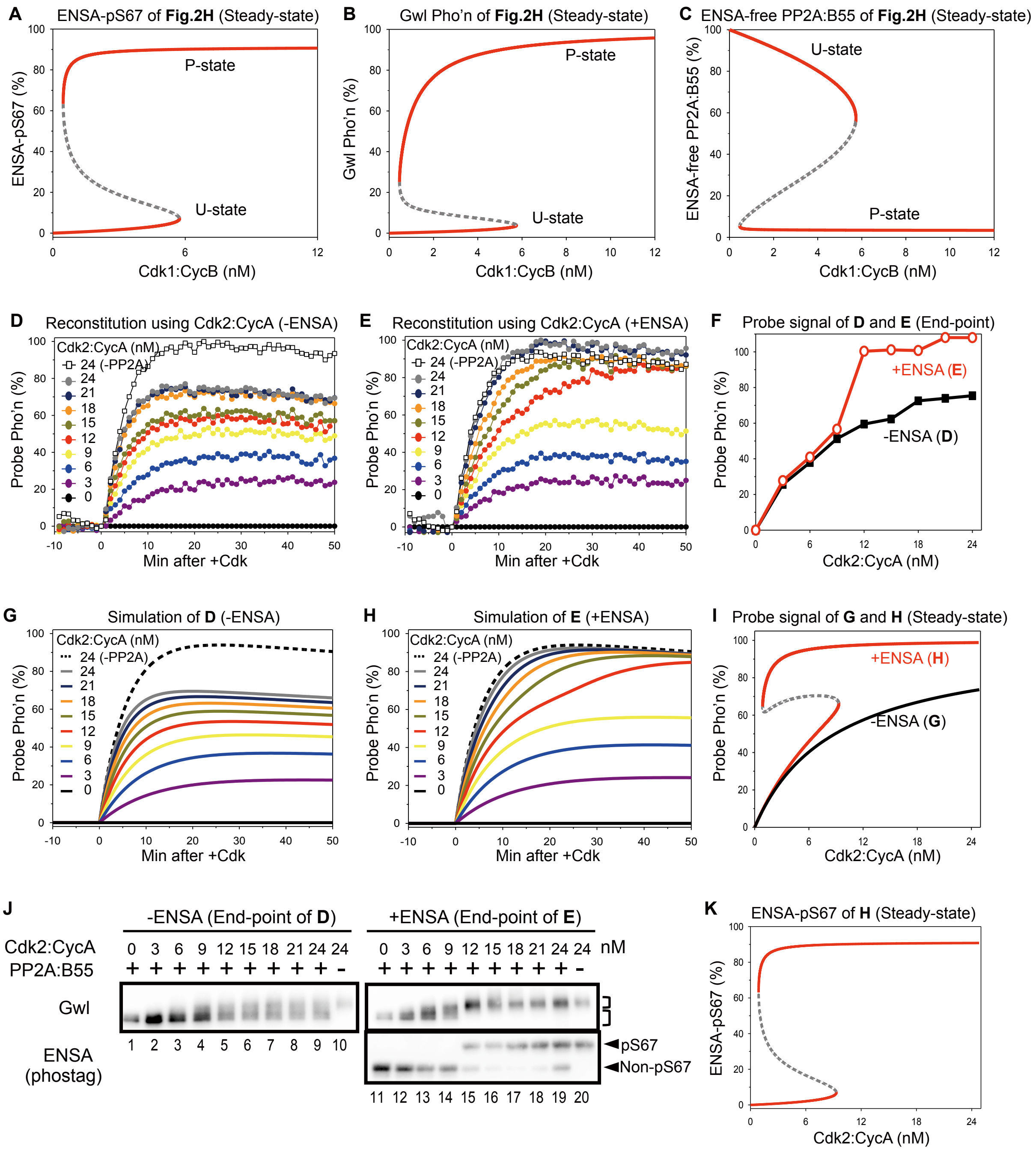

Figure S3 (Related to Figure 2, Tables S1 and S2): Kinetic modeling of the experiment in Figure 2 with Cdk1:CycB (A-C) and reproduction of Figure 2 using Cdk2:CycA complex (D-K). Model-calculated steady states of ENSA-pS67 (**A**), pGwl (**B**) and ENSA-free PP2A:B55 (**C**) are plotted for the experiment on Fig. 2. The edges of the upper and the lower steady states of the one-parameter (Cdk1:CycB) bifurcation diagrams represent Cdk1 activity thresholds where the system undergoes an abrupt state transition between unphosphorylated (U) and phosphorylated (P) states. **(D-K)** Phosphorylation of S50-1G12 probe was induced with indicated concentrations of Cdk2:CycA in the absence (**D** and **G**) and in the presence (**E** and **H**) of ENSA. Note that this probe is 7-times less sensitive to PP2A:B55 than T50-NCP probe as shown in Table S2, resulting in relatively higher phosphorylation level in all samples than those in Fig. 2. Experimental data (**D**, **E**, **F**) and calculations with the model (**G**, **H**, **I**) are shown. Probe phosphorylation is expressed relative to the maximal phosphorylation achieved by omitting PP2A:B55

(lanes 10 and 20 in **J** of this figure, otherwise 50 nM) from the reconstitution system. The end-points of probe phosphorylation (**F**) in the experiments are matched by the stable steady states of the model (**I**). (**J**) Western-blot analysis of Gwl and ENSA phosphorylations at the end-point of panels **D** and **E**. The slower-migrating form of ENSA is Ser67-phosphorylated. (**K**) One-parameter bifurcation diagram for ENSA-pS67. Representative result of 6 experiments is shown here.

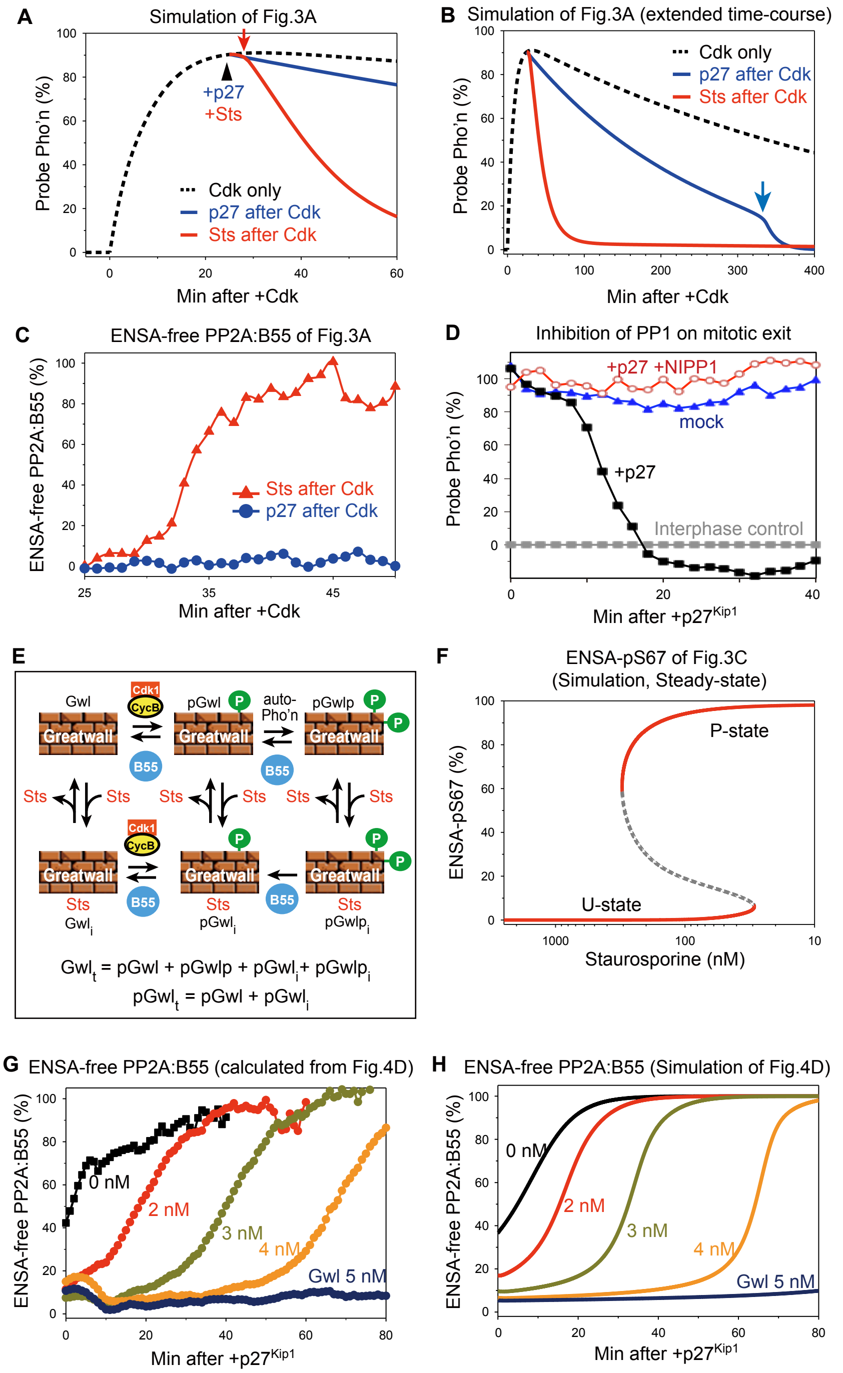

Figure S4 (Panels A-F and G-H are related to Figure 3 and 4, respectively. Also related to Tables S1 and S2): The effect of protein-kinase inhibitors. (**A**) Numerical simulation of protein-kinase inhibition in the P-state (corresponds to Fig. 3A) by p27 (blue curve) and Staurosporine (red curve). Kinase inhibition was implemented at 25 mins (blue and red curves) and Staurosporine concentration was set to 10 μM. (**B**) Extended time-course simulation of **A**. Timings of PP2A:B55 reactivation are indicated by colored arrows in **A** and **B**. (**C**) The release of PP2A:B55 from ENSA inhibition after protein-kinase inhibition by p27 and Staurosporine at 25 mins in the experiment of Fig. 3A. Observe the time-delay in PP2A:B55 release after Staurosporine addition caused by the 'unfair competition' mechanism. (**D**) PP1 activity is required for dephosphorylation of Cdk1 substrate on mitotic exit in *Xenopus* egg extract. (**E**) Wiring diagram to explain the extension of the model to the effect of Staurosporine (Sts) on Gwl. Staurosporine reversibly binds to all forms of Greatwall and thereby inhibits both auto-

phosphorylation and phosphorylation of ENSA. (**F**) Steady state dependence of ENSA S67-phosphorylation on Staurosporine concentration (increasing from the right to left). About ten-times more concentration of Staurosporine is required to induce the P→U transition than to block the U→P transition. (**G, H**) Release of PP2A:B55 from the pENSA:PP2A:B55 complex after Cdk inhibition with p27. (**G**) Calculation of ENSA-free PP2A:B55 from experimental data of probe dephosphorylation in Fig. 4D. The high concentration of Cdk2:CycA complex (100 nM) in this experiment causes significant T28-phosphorylation of ENSA, which reduces the level of ENSA-free PP2A:B55 at 0 min even in the absence of Gwl (black). The time-delay of PP2A:B55 release from ENSA inhibition is longer at higher Gwl concentrations. (H) Model simulations of **G**.

Table S1: Kinetic parameters used in the model. Related to Figures 2, 3, 4, S3, S4 and Table S2.

Parameter	Description	Value
Cdk	Cdk1:CycB or Cdk2:CycA complex	0 – 100 nM
Sub_T	Total concentration of probe	50 nM
$k_{Cdk,Sub}$	Probe phosphorylation by Cdk:Cyc complexes	see Table S2
$k_{B55,Sub}$	Probe dephosphorylation by PP2A:B55	see Table S2
$k_{d,Sub}$	Probe decay for T50-NCP and S50-1G12	0.001 min^{-1} and 0.002 min^{-1}
$ENSA_T$	Total concentration of ENSA	300 nM
$B55_T$	Total concentration of PP2A:B55	50 nM
$k_{Gw,ENSA}$	ENSA phosphorylation by active Greatwall	0.2358 $nM^{-1}min^{-1}$
k_{ass}	Association of pS67-ENSA and PP2A:B55	0.3350 $nM^{-1}min^{-1}$
k_{diss}	Dissociation of the pS67-ENSA:PP2A:B55 complex	0.0267 min^{-1}
k_{cat}	Dephosphorylation of pS67-ENSA by PP2A:B55	2.7504 min^{-1}
Gwl_T	Total concentration of Greatwall	20 nM
$k_{Cdk,Gwl}$	Greatwall phosphorylation by Cdk1:CycB or Cdk2:CycA	0.0023 or 0.0012 $nM^{-1}min^{-1}$
$k_{B55,Gwl}$	Greatwall dephosphorylation by PP2A:B55	0.1447 $nM^{-1}min^{-1}$
α	Equilibrium of Gwl de- and auto-phosphorylation	0.003 nM^{-1}
$k_{Cdk,ENSA}$	ENSA phosphorylation by active Cdk:Cyc complex	0.0016 $nM^{-1}min^{-1}$
$k_{ass,T}$	Association of pT28-ENSA and PP2A:B55	1.6068 $nM^{-1}min^{-1}$
$k_{diss,T}$	Dissociation of pT28-ENSA:PP2A:B55 complex	145.256 min^{-1}
$k_{cat,B55T}$	Dephosphorylation of pT28-ENSA by PP2A:B55	0.5211 min^{-1}

Table S2: Phosphorylation and dephosphorylation rate constants for luminescent probes. Related to Figures 2, 3, 4, S3, S4 and Table S1.

Name of probe	$k_{Cdk,Sub}$ ($nM^{-1}min^{-1}$)	$k_{B55,Sub}$ ($nM^{-1}min^{-1}$)
T50-NCP	0.010	0.0090
S50-1G12	0.0073	0.0013
S50-1G4	0.0235	0.017

Supplemental Experimental Procedures

Interphase *Xenopus* cell-free egg extracts

Cytostatic factor-arrested extract prepared from unfertilized *Xenopus* eggs [S3] was released into interphase for 40 mins at 23°C by addition of $CaCl_2$ (0.4 mM) in the presence of cycloheximide (0.1 mg/mL). Extract was stored in liquid nitrogen.

Immunodepletion

Immunodepletion of ENSA from egg extracts was done as described before, except use of protein-A Sepharose beads [S4].

Antibodies and chemicals

Anti-phosphoSer Cdk targets and -GFP antibodies were purchased from Cell Signaling Technology (#2324) and Medical & Biological Laboratories (clone 1E4), respectively. Anti-phosphoSer50 Fizzy and -phosphoTyr15 Cdc2 antibodies were generous gifts from Drs. Tim Hunt and Julian Gannon (Crick Institute, UK). Anti-Gwl, -ENSA, -phosphoS67-ENSA, and -phosphoT28-ENSA antibodies were previously described [S5, 6]. Coelenterazine-h, PD166285 dihydrochloride, Staurosporine and phos-tag were purchased from Wako (#035-22991), R&D SYSTEMS (#3785/1), LC Laboratories (#S-9300) and NARD (AAL-107), respectively.

Recombinant protein

Polyhistidine-tagged *Xenopus* ENSA, human Cks2, $p27^{Kip1}$, and luminescent probes were expressed and purified from bacteria strain BL21(DE3) codon+(PR) using HisPur Ni-NTA Resin (Thermo Scientific) according to the manufacturer's protocol. GST-tagged *Xenopus* Greatwall was expressed in and purified from Sf9 insect cells as described previously [S7] and GST portion was removed by using HRV-3C protease. The Aα, B55δ, and Cα subunits of PP2A complex were simultaneously expressed in HighFive insect cells and purified as described [S4]. Cdk2:CycA complex was prepared as described [S8]. Cdk1:CycB complex was purified as described [S9] with some modifications as follows. Maltose-binding protein-tagged human CycB protein lacking 172 amino acids of its amino terminus (CycB-ΔN) was expressed and purified from bacteria by using amylose resin (New England Biolab). CycB-ΔN protein was incubated with interphase egg extract in the presence of PD166285 (1 µM) for 30 mins at 23°C. Cdk:Cyc complexes were purified by using Suc1 beads, and eluate from the beads was further purified using amylose resin. All proteins were dialyzed against a storage buffer (20 mM Tris-HCl, 150 mM NaCl, 0.01% Tween-20, 0.1 mM dithiothreitol (DTT), pH7.5) and stocked in small aliquots at -80°C. Unless otherwise mentioned, each protein concentration in the reconstitution system was as follows: Cks2=200 nM, Gwl=20 nM, ENSA=300 nM, PP2A:B55=50 nM. Our rough estimates of endogenous concentrations of these proteins are as follows: Gwl=17~21 nM, ENSA=200~300 nM, PP2A:B55=50~70 nM.

Luminescent probe assay

Luminescent phosphorylation probes, consisting of engineered luminescent protein called "NanoLantern" inserted just after its Gly228 residue with the phosphopeptide-binding WW domain of Pin1 and a modified ~30-amino-acid-long peptide derived from *Xenopus* Fizzy containing its Ser50 residue (Fig. S1A), were developed to detect the ratio of Cdk and PP2A:B55 activities [S1]. We used 3 probes with different phosphorylation sequences as follows: Probe S50-1G4: (N')-RSAYMMGGRRVSANTSTL*SPMKASNRSHSSSGGLE-(C'), S50-1G12: (N')-RSGGGCSSLNTSANTSTL*SPMKASNYSHRNAYELE-(C'), T50-NCP: (N')-RSGGGRAEKKKPANTSTL*TPMKASNTKQAKKGGVE (C'). Asterisk denotes each phosphorylation site. Serine/Threonine-to-Alanine mutants of the probes were also made (Figs S1B, C). Purified probe (50 nM) was mixed with an appropriate combination of other proteins in a reconstitution buffer (20 mM Tris-HCl, 50 mM NaCl, 5 mM $MgCl_2$, 7.5 mM KCl, 10 µM $MnCl_2$, 0.01% Tween-20, 1 mM DTT, 20 mM ascorbic acid, 0.5 mM adenosine triphosphate, 50 µg/mL bovine serum albumin, 10 µM Coelenterazine-h (CTZ), pH7.5). Assays were started in white-wall 96-well microplate (#4ti-0961, 4titude) at 30°C, and luminescence was measured using Infinite F200 Pro microplate reader (TECAN). Assays in egg extracts contained a probe at 200 nM and Coelenterazine-h at 10 µM. All data were standardized by using samples without Cdk in the reconstitution system or without addition of CycB in egg extracts as baseline controls.

SDS-PAGE analyses with phos-tag reagent and quantification of immunoblot data

To quantitate S67-phosphorylated form of ENSA, standard SDS polyacrylamide gel was supplemented with 15 μM of phos-tag reagent, which causes slower migration of phosphorylated proteins on electrophoresis. Under this condition, ENSA phosphorylation on its S67, but not on T28, significantly decreases its mobility and resulted in an up-shifted band [S6]. All immunoblot signals were acquired using a FUSION SOLO S system (VILBER LOURMAT). In Fig.4B, signal intensities of the up-shifted bands were quantified using the Fusion Capt Advance software. For better quantification, a tendency of CDK-phosphorylated ENSA to react less with our anti-ENSA antibody was also considered (Compare the lanes 1 and 2 of Fig.4B).

Mathematical modeling of the Greatwall-ENSA pathway

We used nonlinear ordinary differential equations (ODEs) to describe the dynamics of the Gwl-ENSA pathway. The ODEs describe the time-rate of change of the components in the pathway using the law of mass action for each elementary reaction. Each enzymatic reaction (except pENSA dephosphorylation by PP2A:B55) is approximated as a bimolecular, second-order reaction, thereby ignoring the enzyme-substrate complexes. This approximation is reasonable if the Michaelis-constants (K_M) of the enzymes are larger than the substrate concentrations, in which case the reaction is always first-order for the substrate. This assumption is experimentally validated for phosphorylation and dephosphorylation of the probes with purified Cdk:cyclin and PP2A:B55 complexes, respectively. This simplifying assumption had to be waived in the case of pENSA dephosphorylation by PP2A:B55 because of their tight binding (small K_M value).

Cdk:cyclin complex phosphorylates Gwl at site T193 (pGwl), which activates the intramolecular autophosphorylation of Gwl into its double-phosphorylated form:

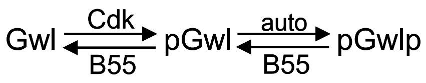

Both phosphorylation reactions are counter-acted by PP2A:B55 (B55)-catalysed dephosphorylations, because PP2A:B55 is the only phosphatase in the system. Since the total Gwl concentration (Gwl_T) is constant (conservation condition), Gwl dynamics can be described by two ODEs:

$$\frac{d[pGwl]_t}{dt} = k_{Cdk,Gw} \cdot [Cdk] \cdot ([Gwl]_T - [pGwl]_t) - k_{B55,Gw} \cdot [B55] \cdot ([pGwl]_t - [pGwlp]) \qquad (1')$$

$$\frac{d[pGwlp]}{dt} = k_{auto} \cdot ([pGwl]_t - [pGwlp]) - k'_{B55,Gw} \cdot [B55] \cdot [pGwlp] \qquad (2)$$

where pGwlt is the sum of the two phosphorylated forms (pGwlt = pGwl + pGwlp). Our parameter estimation suggests that the second ODE is very fast, therefore we assume that pGwl and pGwlp are in pseudo-steady states:

$$[pGwlp] = \frac{[pGwl]_t}{1+\alpha \cdot [B55]} \qquad [pGwl] = = \frac{\alpha \cdot [B55] \cdot [pGwl]_t}{1+\alpha \cdot [B55]} \qquad (3)$$

where α indicates the ratio of $k'_{B55,Gw}$ and k_{auto} rate constants of the second step. With this simplification, Eq.(1') takes the following form:

$$\frac{d[pGwl]_t}{dt} = k_{Cdk,Gw} \cdot [Cdk] \cdot ([Gwl]_T - [pGwl]_t) - k_{B55,Gw} \cdot \alpha \cdot [B55]^2 \cdot \frac{[pGwl]_t}{1+\alpha \cdot [B55]} \qquad (1)$$

In the model, ENSA is phosphorylated at the S67 site (pENSA) by auto-phosphorylated Greatwall-kinase (pGwlp), which is the only form that catalyses ENSA phosphorylation. pENSA binds rapidly and tightly to free PP2A:B55, which dephosphorylates it:

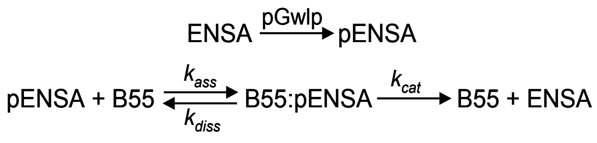

Considering that total ENSA concentration ($[ENSA]_T$) in our reconstituted system is constant, we derive an ODE for the sum of the two phosphorylated forms ($[pENSA]_t$ = [pENSA] + [B55:pENSA]):

$$\frac{d[pENSA]_t}{dt} = k_{Gw,ENSA} \cdot [pGwlp] \cdot ([ENSA]_T - [pENSA]_t) - k_{cat,B55} \cdot [B55{:}pENSA] \qquad (4)$$

and a second ODE for PP2A:B55-pENSA complex:

$$\frac{d[B55:pENSA]}{dt} = k_{ass} \cdot [B55] \cdot ([pENSA]_t - [B55:pENSA]) - (k_{cat,B55} + k_{diss}) \cdot [B55:pENSA] \quad (5)$$

The free PP2A:B55 (B55) concentration is calculated from the conservation condition that the total concentration ($B55_T$) is constant:

$$[B55] = [B55]_T - [B55:pENSA] \quad (6)$$

Finally, the probe (Sub) carrying a Fizzy phosphorylation site is interconverted by Cdk:cyclin and PP2A:B55 complexes between phosphorylated (pSub) and unphosphorylated forms (Sub_T – pSub):

$$\frac{d[pSub]}{dt} = k_{Cdk,Sub} \cdot [Cdk] \cdot ([Sub]_T - [pSub]) - \left(k_{B55,Sub} \cdot [B55] + k_{d,Sub}\right) \cdot [pSub] \quad (7)$$

Although the total concentration of the probe is constant, it slowly loses its light emittance, which we approximate with a slow exponential decay ($k_{d,Sub}$):

$$\frac{d[Sub]_T}{dt} = -k_{d,Sub} \cdot [Sub]_T \quad (8)$$

Cdk2 inhibition by p27 is simulated by setting the Cdk parameter to zero, because the inhibitor is used in large excess (> 450 nM) over the kinase.

This system of differential- and algebraic-equations which was solved by numerical integration, provides a comprehensive picture of temporal changes in our reconstitution experiments. The temporal evolution of the biochemical system leads to a steady state which corresponds to the end-point of the experiments. The steady state solutions of the system are conveniently characterized by bifurcation diagrams, where one of the dynamical variables is plotted on the y-axis as a function of Cdk:cyclin complex, which is our experimentally controlled parameter.

The following code can be used with freely available XPPAut software (http://www.math.pitt.edu/~bard/xpp/xpp.html) to reproduce our figures of temporal simulations (Fig. 2G,H, 4C, S3G,H, and S4H). Since ENSA becomes phosphorylated directly by Cdk:cyclin complexes at the T28 site, our code also includes this reaction, which becomes significant when the ratio of Cdk:cyclin to Gwl is very large. The two independent phosphorylations result in three different phosphorylated forms of ENSA (pENSA, ENSAp and pENSAp) indicated on the left (S67) and on the right (T28), respectively. T28-ENSA phosphorylation is also removed by PP2A:B55, at least in our system.

```
# .ode file for time-course simulation of the reconstituted system
dSubT/dt = - kdSub*SubT
dpSub/dt =  kCdkSub*Cdk*(SubT-pSub) - (kB55Sub*B55 +  kdSub)*pSub
dpENSA/dt = kGwENSA*pGwlp*ENSA + kdiss*B55pENSA - kass*pENSA*B55 - kCdkENSA*Cdk*pENSA
+ kcatB55T*pENSApB55
dB55pENSA/dt = kass*pENSA*B55 - kdiss*B55pENSA - kcatB55*B55pENSA
dpENSAp/dt = kCdkENSA*Cdk*pENSA + kdissT*pENSApB55 - kassT*pENSAp*B55 +
kGwENSA*pGwlp*ENSAp + kdiss*B55pENSAp - kass*B55*pENSAp
dpENSApB55/dt = kassT*pENSAp*B55 - kdissT*pENSApB55 - kcatB55T*pENSApB55
dB55pENSAp/dt = kass*pENSAp*B55 - kdiss*B55pENSAp - kcatB55*B55pENSAp
dENSAp/dt = kcatB55*B55pENSAp - kassT*ENSAp*B55 + kdissT*ENSApB55 - kGwENSA*pGwlp*ENSAp
+ kCdkENSA*Cdk*ENSA
dENSApB55/dt = kassT*B55*ENSAp - kdissT*ENSApB55 - kcatB55T*ENSApB55
dpGwlt/dt = kCdkGw*Cdk*(GwlT - pGwlt) - kB55Gw*alfa*B55^2*pGwlt/(1 + alfa*B55)
pGwlp = pGwlt/(1+alfa*B55)
B55 = B55T - B55pENSA - pENSApB55 - B55pENSAp - ENSApB55
ENSA = ENSAtot - pENSA - B55pENSA - pENSAp - pENSApB55 - B55pENSAp - ENSAp - ENSApB55
aux S67pENSA = pENSA + B55pENSA + pENSAp + B55pENSAp + pENSApB55
aux pGwlp = pGwlt/(1 + alfa*B55)
aux B55free = B55T - B55pENSA - pENSApB55 - B55pENSAp - ENSApB55
init SubT=50, pSub=0, pGwlt=0

par Cdk=0
# kinetic parameters for Cdk1:CycB and T50-NCP probe:
par kCdkSub=0.01, kB55Sub=0.009, kdSub=0.001
par ENSAtot=300, B55T=50, kGwENSA=0.2358
par kass=0.3350, kdiss=0.0267, kcatB55=2.7504
par GwlT=20, kCdkGw=0.0023, kB55Gw=0.1447, alfa=0.003
par kCdkENSA=0.0016, kassT=1.6068, kdissT=145.256, kcatB55T=0.5211
# for Cdk2:CycA and S50-1G12 probe set:
# kCdkSub=0.0073, kB55Sub=0.0013, kdSub=0.002, kCdkGw=0.0012
@ total=50,dt=0.5,meth=STIFF,xp=time,yp=pSub,xlo=0,xhi=50,ylo=0,yhi=50
@ NTST=15,NMAX=1000000,NPR=10000,DS=0.01,BOUNDS=2000
@ DSMAX=0.02,DSMIN=0.002,PARMIN=0,PARMAX=25
@ AUTOXMIN=0,AUTOXMAX=25,AUTOYMIN=0,AUTOYMAX=50
done
```

To calculate bifurcation diagrams (Figs 2I, 4A, S3A-C and S3I, K), dynamic variable of Sub_T in the code has to be made as a constant parameter with a value of 50 nM, in order to have a steady state for the probe.

The model with Staurosporine effect

Staurosporine is a competitive inhibitor of ATP binding to protein-kinases, therefore it blocks the activity of both Cdk and Gwl. In our model, Staurosporine binds with the same affinity to both Cdk and all three forms of Gwl kinase, thereby inhibiting their kinase activities including auto-phosphorylation of Gwl (Fig. S4E). The model is applicable at low Staurosporine concentration as well because it does not assume constant free inhibitor concentration. The following XPPAut code can be used to simulate Staurosporine effect on Figs. S4A,B.

```
# .ode file for time-course simulation of the Staurosporine effect
dSubT/dt = - kdSub*SubT
dpSub/dt =  kCdkSub*Cdka*(SubT-pSub) - (kB55Sub*B55 +  kdSub)*pSub
dCdka/dt = kdisSts*(Cdk - Cdka) - kasSts*Cdka*(10^logStau - (Cdk - Cdka) - (Gwltot - Gwlfree))
dpENSA/dt = kGwENSA*pGwlp*ENSA + kdiss*B55pENSA - kass*pENSA*B55 - kCdkENSA*Cdka*pENSA
+ kcatB55T*pENSApB55
dB55pENSA/dt = kass*pENSA*B55 - kdiss*B55pENSA - kcatB55*B55pENSA
dpENSAp/dt = kCdkENSA*Cdka*pENSA + kdissT*pENSApB55 - kassT*pENSAp*B55 +
kGwENSA*pGwlp*ENSAp + kdiss*B55pENSAp - kass*B55*pENSAp
dpENSApB55/dt = kassT*pENSAp*B55 - kdissT*pENSApB55 - kcatB55T*pENSApB55
dB55pENSAp/dt = kass*pENSAp*B55 - kdiss*B55pENSAp - kcatB55*B55pENSAp
dENSAp/dt = kcatB55*B55pENSAp - kassT*ENSAp*B55 + kdissT*ENSApB55 - kGwENSA*pGwlp*ENSAp
+ kCdkENSA*Cdka*ENSA
dENSApB55/dt = kassT*B55*ENSAp - kdissT*ENSApB55 - kcatB55T*ENSApB55
dGwlt/dt =  kCdkGw*Cdka*(Gwltot - Gwlt) - kB55Gw*B55*alfa*B55*Gwlt/(alfa*B55 + Gwlfree/Gwltot)
dGwlfree/dt = kdisSts*(Gwltot - Gwlfree) - kasSts*Gwlfree*(10^logStau - (Cdk - Cdka) - (Gwltot - Gwlfree))
pGwlt = alfa*B55*Gwlt/(alfa*B55 + Gwlfree/Gwltot)
pGwlp = Gwlfree*(Gwlt - pGwlt)/Gwltot
B55 = B55T - B55pENSA - pENSApB55 - B55pENSAp - ENSApB55
ENSA = ENSAtot - pENSA - B55pENSA - pENSAp - pENSApB55 - B55pENSAp - ENSAp - ENSApB55
aux S67pENSA = pENSA + B55pENSA + pENSAp + B55pENSAp + pENSApB55
aux pGwlp = pGwlt/(1 + alfa*B55)
aux B55 = B55T - B55pENSA - pENSApB55 - B55pENSAp - ENSApB55
init SubT=50, pSub=0, Cdka=20, Gwlfree=20

par logStau=-10, Cdk=20, kdisSts=0.015, kasSts=0.0001
par kCdkSub=0.0073, kB55Sub=0.0013, kdSub=0.002
par ENSAtot=300, B55T=50, kGwENSA=0.2358
par kass=0.3350, kdiss=0.0267, kcatB55=2.7504
par Gwltot=20, kCdkGw=0.0012, kB55Gw=0.1447, alfa=0.003
par kCdkENSA=0.0016, kassT=1.6068, kdissT=145.256, kcatB55T=0.5211

@ total=25,dt=1,meth=STIFF,xp=time,yp=pSub,xlo=0,xhi=60,ylo=0,yhi=50
@ NTST=50,NMAX=100000000,NPR=1000000,DS=0.01,BOUNDS=2000
@ DSMAX=0.01,DSMIN=0.001,PARMIN=0,PARMAX=3.5
@ AUTOXMIN=1,AUTOXMAX=2.5,AUTOYMIN=0,AUTOYMAX=50
done
```

To calculate bifurcation diagrams (Fig. 3C, Fig. S4F), dynamic variable of Sub_T in the code has to be made as a constant parameter with a value of 50 nM, in order to have a steady state for the probe.

Estimation of the kinetic parameters of the model

Although the concentrations within the reconstituted system are known, the model also contains numerous kinetic parameters whose values have to be estimated. $k_{Cdk,Sub}$ was determined by phosphorylation of the probe with different concentrations of Cdk2:CycA complex in the absence of PP2A:B55, in which case the phosphorylated (pSub) probe follows the time course:

$$[pSub] = [Sub]_T \cdot \left(1 - e^{-k_{Cdk,Sub} \cdot [Cdk] \cdot t}\right) \tag{9}$$

The Cdk dependence of the specific phosphorylation rate:

$$\frac{1}{[Sub]} \frac{d[Sub]}{dt} = -k_{Cdk,Sub} \cdot [Cdk] \tag{10}$$

provides $k_{Cdk,Sub}$. In the presence of PP2A:B55, the probe phosphorylation rapidly reaches a steady state ($pSub_{ss}$), whose value is hyperbolically dependent on Cdk concentration:

$$[pSub]_{ss} = \frac{[Sub]_T \cdot k_{Cdk,Sub} \cdot [Cdk]}{k_{B55,Sub} \cdot [B55] + k_{Cdk,Sub} \cdot [Cdk]} \tag{11}$$

which can be linearized similarly to the Lineweaver-Burk plot of the Michaelis-Menten equation:

$$\frac{1}{[pSub]_{ss}} = \frac{1}{[Sub]_T} + \frac{k_{B55,Sub} \cdot [B55]}{[Sub]_T \cdot k_{Cdk,Sub}} \cdot \frac{1}{[Cdk]} \tag{12}$$

and the slope of the line provides the value of $k_{B55,Sub}$. An alternative estimate of $k_{B55,Sub}$ is provided by the exponential decay of the phosphorylated probe after p27 addition.

The kinetic parameters of ENSA and PP2A:B55 interactions were already characterized [S10] with $K_M \cong 1$ nM and $k_{cat} = 3$ min^{-1}. These values were used as initial guesses in the search of the values of the remaining parameters using Matlab routine MEIGO [S11]. The kinetic parameters of the model are summarized in Table S1.

Estimation of the concentration of free PP2A:B55 from the rate of dephosphorylation of the probe

Figs. S4C, S4G illustrate the release of PP2A:B55 from the complex with pENSA after p27 inhibition of Cdk or Staurosporine inhibition of both protein kinases. Assuming complete block of probe phosphorylation by high concentration of kinase inhibitors, the specific rate of change for phosphorylated probe (pSub) is given:

$$\frac{1}{[pSub]} \frac{d[pSub]}{dt} = -k_{B55,Sub} \cdot [B55]$$

The specific rate of change of [pSub] was estimated by fitting a second-order polynomial to odd number (N=15, 17 or 19) experimental values of pSub (in nM) using the method of least squares and dividing the first derivative of the fitted curve by [*pSub*] at the central point. This procedure was repeated for successive central points and dividing the specific rates by the constant value of $k_{B55,Sub}$ provided the B55 time-course.

Supplemental References

S1. Saito, K., Chang, Y.F., Horikawa, K., Hatsugai, N., Higuchi, Y., Hashida, M., Yoshida, Y., Matsuda, T., Arai, Y., and Nagai, T. (2012). Luminescent proteins for high-speed single-cell and whole-body imaging. Nat Commun *3*, 1262.

S2. Blake-Hodek, K.A., Williams, B.C., Zhao, Y., Castilho, P.V., Chen, W., Mao, Y., Yamamoto, T.M., and Goldberg, M.L. (2012). Determinants for activation of the atypical AGC kinase Greatwall during M phase entry. Molecular and cellular biology *32*, 1337-1353.

S3. Murray, A.W., and Kirschner, M.W. (1989). Cyclin synthesis drives the early embryonic cell cycle. Nature *339*, 275-280.

S4. Mochida, S., Ikeo, S., Gannon, J., and Hunt, T. (2009). Regulated activity of PP2A-B55 delta is crucial for controlling entry into and exit from mitosis in Xenopus egg extracts. Embo J *28*, 2777-2785.

S5. Mochida, S., Maslen, S.L., Skehel, M., and Hunt, T. (2010). Greatwall phosphorylates an inhibitor of protein phosphatase 2A that is essential for mitosis. Science *330*, 1670-1673.

S6. Mochida, S. (2014). Regulation of alpha-endosulfine, an inhibitor of protein phosphatase 2A, by multisite phosphorylation. FEBS J *281*, 1159-1169.

S7. Castilho, P.V., Williams, B.C., Mochida, S., Zhao, Y., and Goldberg, M.L. (2009). The M phase kinase Greatwall (Gwl) promotes inactivation of PP2A/B55delta, a phosphatase directed against CDK phosphosites. Mol Biol Cell *20*, 4777-4789.

S8. Brown, N.R., Noble, M.E., Endicott, J.A., Garman, E.F., Wakatsuki, S., Mitchell, E., Rasmussen, B., Hunt, T., and Johnson, L.N. (1995). The crystal structure of cyclin A. Structure *3*, 1235-1247.

S9. Kusubata, M., Tokui, T., Matsuoka, Y., Okumura, E., Tachibana, K., Hisanaga, S., Kishimoto, T., Yasuda, H., Kamijo, M., Ohba, Y., et al. (1992). p13suc1 suppresses the catalytic function of p34cdc2 kinase for intermediate filament proteins, in vitro. J Biol Chem *267*, 20937-20942.

S10. Williams, B.C., Filter, J.J., Blake-Hodek, K.A., Wadzinski, B.E., Fuda, N.J., Shalloway, D., and Goldberg, M.L. (2014). Greatwall-phosphorylated Endosulfine is both an inhibitor and a substrate of PP2A-B55 heterotrimers. eLife *3*, e01695.

S11. Egea, J.A., Henriques, D., Cokelaer, T., Villaverde, A.F., MacNamara, A., Danciu, D.P., Banga, J.R., and Saez-Rodriguez, J. (2014). MEIGO: an open-source software suite based on metaheuristics for global optimization in systems biology and bioinformatics. BMC Bioinformatics *15*, 136.
